# BioEdge-RGC: Compact Neural Policy Transfer from Model-Predictive Control in a Transcriptomics-Informed Optic Nerve Injury Simulation

**DOI:** 10.3390/brainsci16090941

**Published:** 2026-09-02

**Authors:** Roxana Irina Iancu, Lucian Eva, Călin Gheorghe Buzea, Florin Nedeff, Valentin Nedeff, Diana Mirilă, Mirela Panainte-Lehaduș, Maricel Agop, Irina Grădinaru, Dragoș Petru Teodor Iancu

**Affiliations:** 1 Department of Oral Pathology, “Grigore T. Popa” University of Medicine and Pharmacy, 700115 Iași, Romania; roxana.iancu@umfiasi.ro (R.I.I.); dt_iancu@yahoo.com (D.P.T.I.); 2 Department of Oncology and Radiotherapy, “Grigore T. Popa” University of Medicine and Pharmacy, 700115 Iași, Romania; 3 Department of Clinical Laboratory, “Sfântul Spiridon” Emergency Hospital, 700111 Iași, Romania; 4 “Prof. Dr. Nicolae Oblu” Clinical Emergency Hospital, 700309 Iași, Romania; elucian73@yahoo.com (L.E.); calinb2003@yahoo.com (C.G.B.); 5 Faculty of Medicine and Pharmacy, “Dunărea de Jos” University of Galați, 800008 Galați, Romania; 6 National Institute of Research and Development for Technical Physics—IFT Iasi, 700050 Iași, Romania; 7 Department of Environmental Engineering, Mechanical Engineering and Agritourism, Faculty of Engineering, “Vasile Alecsandri” University of Bacău, 600115 Bacău, Romania; florin_nedeff@ub.ro (F.N.); vnedeff@ub.ro (V.N.); mirelap@ub.ro (M.P.-L.); m.agop@yahoo.com (M.A.); 8 Department of Implantology, Removable Dentures, Technology, Discipline of Dental Materials, Faculty of Dental Medicine, “Grigore T. Popa” University of Medicine and Pharmacy, 700115 Iași, Romania; irina.gradinaru@umfiasi.ro; 9 Department of Radiotherapy, Regional Institute of Oncology, 700483 Iași, Romania

**Keywords:** retinal ganglion cells, optic nerve crush, model predictive control, compact policy learning, regional edge inference, knowledge distillation, single-cell transcriptomics, computational neuroscience, missing observations, communication compression

## Abstract

Background/Objectives: Optic nerve injury involves heterogeneous neuronal and microenvironmental responses that may require sequential, state-dependent intervention. BioEdge-RGC was developed as a transcriptomics-informed computational benchmark for transferring a globally planned model-predictive-control policy to compact regional neural models operating with incomplete observations and reduced communication. Methods: Public mouse optic-nerve-crush transcriptomic datasets were summarized into seven retinal ganglion cell modules and eight retinal environment modules and fused into a 15-dimensional temporal reference state for a 16-region, five-step simulator. A candidate-constrained MPC expert was approximated by a central neural teacher and local, neighbour-aware, and coordinated regional students trained using hybrid knowledge distillation or direct MPC supervision. Missing observations, severe simulated injury, INT8 quantization, local parameter perturbations, and an independent GSE229033 transcriptomic plausibility assessment were evaluated. Results: The central teacher retained 99.90% of the MPC objective, and the coordinated distilled student retained 99.69% of teacher performance while reducing modeled communication from 5520 to 1080 bytes per episode. The coordinated-versus-local mean-objective difference was +0.000053 and did not meet the predefined +0.008 threshold; direct MPC supervision performed at least as well as hybrid distillation. The principal objective-based conclusions were preserved across all nine local parameter conditions, whereas the coordinated failure-rate advantage was not parameter-robust. In GSE229033, six of seven modules changed in the predefined favorable direction, but the oriented composite bootstrap interval included zero. Conclusions: Compact regional neural policies reproduced MPC performance with minimal objective loss and substantially lower modeled communication. The external transcriptomic analysis provided limited plausibility support for the RGC state orientation, while neither analysis validated simulator dynamics, controller efficacy, biological treatment effects, or clinical applicability.

## 1. Introduction

Adult mammalian central nervous system neurons have a limited capacity to regenerate injured axons. This limitation is particularly evident in the optic nerve, where axonal injury is followed by progressive retinal ganglion cell (RGC) dysfunction, cell death, and incomplete spontaneous regeneration. Experimental manipulation of intrinsic growth pathways, including PTEN/mTOR and SOCS3/JAK–STAT signaling, can promote RGC survival and axonal regrowth [1,2,3]. However, even potent interventions do not uniformly restore the complete sequence required for functional repair. Neuronal survival, axon initiation, lesion crossing, directed extension, target reconnection, synaptic stabilization, and functional maturation represent distinct biological objectives that may require different interventions and temporal schedules [4].

The response to optic nerve injury is also markedly heterogeneous. Single-cell transcriptomic profiling after optic nerve crush has demonstrated that molecularly distinct RGC types differ substantially in their susceptibility to degeneration and in the transcriptional programs activated before survival or cell death. The GSE137398 study followed injured mouse RGCs across a longitudinal series extending from the uninjured state to 14 days after optic nerve crush and identified gene-expression patterns associated with differential resilience [1]. These data provide an opportunity to represent injury progression using biologically interpretable state variables rather than a single aggregated measure of neuronal damage [1,5].

RGC fate is not determined exclusively by cell-intrinsic mechanisms. Müller glia, astrocytes, microglia, vascular cells, and extracellular matrix components undergo their own injury-associated transitions and modify the environment in which RGC survival and regeneration occur. Whole-retina single-cell analysis associated with GSE241268 characterized extensive post-injury changes in non-RGC populations and identified cell–cell communication patterns linking injured RGCs to the surrounding retinal environment. That study also identified the μ-opioid receptor Oprm1 as a candidate neuroprotective factor and reported improved RGC survival following RGC-specific Oprm1 overexpression [6]. These findings support a computational representation that combines neuronal state with glial, inflammatory, vascular, and extracellular matrix information rather than modeling RGCs in isolation.

The temporal and spatial heterogeneity of optic nerve injury creates a sequential control problem. Adjacent tissue regions may occupy different injury states, and an intervention that is protective during acute cellular stress may become ineffective or detrimental after the biological state has changed. Moreover, realistic control strategies are constrained by incomplete measurements, limited intervention capacity, uncertain treatment responses, and the need to balance neuronal protection, inflammatory stabilization, and regenerative support. Fixed schedules and spatially uniform actions cannot explicitly represent these constraints. A computational control framework should therefore account for regional state, spatial propagation, action timing, intervention burden, missing observations, and the accumulation of partially irreversible injury.

Several control strategies could in principle be considered for such a sequential problem. Fixed or open-loop schedules are computationally inexpensive but cannot adapt intervention timing or regional allocation to changing injury states. Classical feedback approaches such as proportional–integral–derivative or linear-quadratic control are attractive when dynamics can be represented adequately by low-order or approximately linear models, but they are less naturally suited to discrete regional actions, state-dependent penalties, and explicit intervention-budget constraints. Robust and adaptive control can address uncertainty in model parameters, whereas dynamic programming and reinforcement-learning approaches can accommodate nonlinear sequential decisions but may require either exhaustive state–action evaluation or extensive interaction data and may provide less direct enforcement of hard constraints [7]. The relevant selection criteria therefore include the degree of nonlinearity and state dependence, the need to enforce explicit action and resource constraints, the availability of a predictive system model, real-time computational requirements, and the amount of data available for learning a policy [8,9].

Model predictive control (MPC) was selected as the reference planning framework because it provides a practical compromise among these requirements. At each decision point, MPC evaluates the projected consequences of feasible interventions over a finite future horizon, applies the currently preferred action, and repeats the optimization after the system state has been updated [8]. This receding-horizon formulation permits explicit consideration of state-dependent intervention timing, competing short- and longer-term objectives, and a predefined regional intervention budget. It is also suitable for the present simulator because a predictive transition model is available and the admissible action set can be constrained directly during planning. Importantly, MPC was not assumed to be universally superior to alternative control strategies or globally optimal in the present implementation. Rather, it provided an interpretable, constraint-aware reference policy against which compact learned controllers could be evaluated. Its principal disadvantage in this setting is computational and informational: a globally informed MPC controller requires access to the complete simulated tissue state and repeated evaluation of multiple candidate regional action allocations [7,8]. These requirements motivate the subsequent policy-transfer problem investigated in BioEdge-RGC—whether the behavior of a globally informed planner can be approximated by substantially smaller regional neural models operating with incomplete observations and restricted communication.

Edge artificial intelligence addresses an analogous engineering problem by moving compact inference models closer to local sensors and actuators. Edge execution can reduce the volume of data transmitted to a central processor, decrease dependence on continuous connectivity, and permit local decisions under restricted computational and communication resources [10]. Translating this principle to computational tissue control raises an important question: whether a policy generated using complete global information can be transferred to small regional agents that operate using local observations and compressed messages.

Knowledge distillation provides one mechanism for performing such a transfer. A compact student model is trained not only from hard reference labels but also from the probability distributions, priorities, or internal decision structure generated by a larger teacher model [11,12]. Distillation has primarily been developed for predictive classification and model compression [12]. In a distributed control setting, however, preserving classification accuracy alone is insufficient. The student must also rank competing regions under a global intervention budget, tolerate missing measurements, preserve closed-loop behavior over multiple sequential decisions, and operate with substantially lower communication requirements.

Existing computational studies of optic nerve injury have predominantly focused on transcriptomic characterization, molecular target identification, or modeling of individual biological mechanisms. Edge-AI studies have primarily focused on local inference and communication-efficient deployment [10], whereas knowledge-distillation studies have largely addressed model compression and predictive knowledge transfer [11,12]. Related work on policy distillation and neural approximation or imitation of control policies has demonstrated that compact learned models can reproduce aspects of more computationally demanding reference controllers [13,14,15]. However, the sequential control of a spatially heterogeneous biological injury state under incomplete sensing and constrained communication remains comparatively unexplored. It therefore remains unclear whether a globally planned tissue-control policy can be compressed into small regional agents without a substantial loss of closed-loop performance. A related uncertainty concerns the biological and computational robustness of such a framework. Transcriptomics-derived state modules may reflect idiosyncrasies of the datasets used to construct them unless their orientation is examined in an independent perturbation dataset. Likewise, controller comparisons obtained under a single simulator parameterization may depend on the assumed action effects, propagation strength, process-noise magnitude, or failure definition. Independent transcriptomic plausibility assessment and local parameter-sensitivity analysis therefore provide useful complementary checks, provided that they are not interpreted as biological treatment validation or as global validation of the simulator.

The present study introduces BioEdge-RGC as a computational framework for evaluating whether a multi-step policy generated using complete simulated tissue information can be approximated by compact regional neural models operating with incomplete observations and reduced communication. Public mouse optic-nerve-crush transcriptomic datasets were used to construct a 15-dimensional state representation comprising seven RGC-intrinsic modules and eight retinal environment modules. The RGC component was derived from officially annotated optic-nerve-crush RGCs aggregated at the biological-sample level, whereas the environmental component represented Müller glial, microglial, inflammatory, vascular, and extracellular matrix responses. Because the source datasets originated from different animals and experimental preparations, their integration was restricted to matched temporal anchors and was not interpreted as a paired, multimodal, or causal cell–cell dataset. The seven RGC modules were additionally transferred without modification to the independent GSE229033 Dnmt3a-perturbation dataset to assess their external transcriptomic plausibility. A separate one-factor-at-a-time analysis examined whether the principal comparative controller conclusions depended narrowly on selected simulator coefficients.

A five-step, 16-region simulator was constructed to represent heterogeneous simulated injury severity, directional propagation, accumulated loss of RGC identity, incomplete sensing, and a limited regional intervention budget. A candidate-constrained, receding-horizon MPC reference controller generated action allocations. A central neural teacher was trained to approximate the MPC policy, after which local, neighbour-aware, and coordinated regional students were developed using ranking-aware hybrid distillation. A coordinated student with the same architecture was also trained directly from MPC labels to distinguish the effect of distillation from that of compact behavioural cloning. Missing measurements were handled using explicit availability masks and median imputation, while neighbour and global summaries were represented through a compact INT8 communication codec. The resulting policies were incorporated into the BioEdge-RGC interactive research application.

The methodological novelty lies in evaluating ranked, budget-constrained policy transfer under the simultaneous constraints of spatial heterogeneity, incomplete observations, compressed nonlocal information, closed-loop sequential control, and transcriptomics-derived state coordinates. Rather than using neural networks solely for classification or outcome prediction, BioEdge-RGC examines whether compact regional models can reproduce the sequential allocation behavior of a globally informed planning controller while retaining only limited local or compressed nonlocal information. A further contribution is the architecture-matched comparison between hybrid teacher–student distillation and direct supervision from MPC-generated labels, which separates the value of compact policy learning from any specific benefit attributable to distillation.

Scientifically, this framework provides a controlled way to investigate how much spatial information and inter-regional communication are required to preserve a globally planned policy in a heterogeneous biological-state simulation, and how that requirement changes under missing observations and simulator perturbations. The potential translational relevance is more limited and prospective: if future experimentally calibrated tissue models demonstrate comparable behavior, compact regional control could become relevant to distributed sensing-and-actuation platforms in biological experimentation or medical simulation. The present results, however, do not establish biological treatment efficacy, patient-specific prediction, clinical decision support, or a validated digital twin.

**Figure 1:**
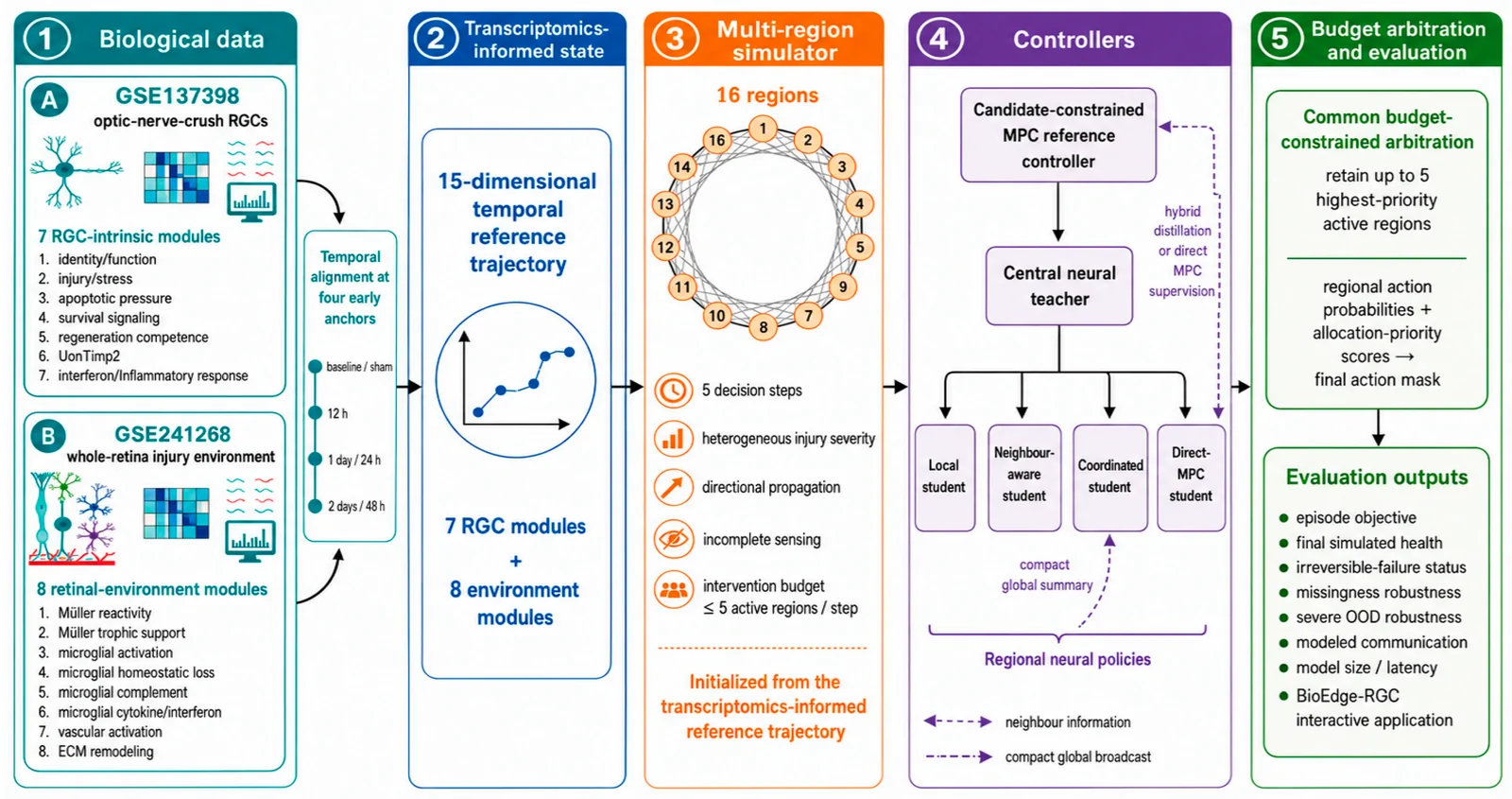
BioEdge-RGC study design and regional policy architecture. Public optic-nerve-crush transcriptomic datasets were summarized into seven RGC-intrinsic and eight retinal environment modules and aligned at four early temporal anchors. The resulting 15-dimensional reference trajectory was used to initialize a five-step simulator containing 16 interconnected regions. A candidate-constrained MPC reference controller generated regional action allocations, which were approximated by a central neural teacher and compact local, neighbour-aware, coordinated, and direct-MPC regional policies. Each neural model produced regional action probabilities and allocation-priority scores. For neural-policy execution, a common budget-arbitration layer assigned active interventions to the five regions with the highest allocation-priority scores at each decision step. Dashed arrows denote compact neighbour or tissue-level information, whereas solid arrows denote state, prediction, and final-action flow.

Accordingly, the aim of this study was to determine whether a globally informed, candidate-constrained MPC policy could be transferred to compact regional neural controllers while preserving closed-loop performance under incomplete sensing and restricted communication. To address this aim, three prespecified research questions were evaluated. First, could a central neural approximation and compact regional policies retain the closed-loop objective of the candidate-constrained MPC reference controller? Second, did neighbour information or a compact global summary provide a meaningful advantage over local observations alone, defined by a coordinated-versus-local mean-objective difference of at least 0.008 and a relative failure reduction of at least 15%? Third, did hybrid teacher–student distillation provide an advantage over direct supervision using the same MPC-generated labels? The primary study workflow, including transcriptomic state construction, multi-region simulation, neural policy transfer, budget-constrained arbitration, and evaluation, is summarized in Figure 1. The primary contribution is therefore the evaluation of compact policy transfer, information requirements, robustness, and modeled communication efficiency within a transcriptomics-informed simulation. The transcriptomic data informed the representation and temporal organization of injury states, whereas intervention effects and state-transition dynamics remained explicit engineering assumptions and did not constitute evidence of optic nerve repair or treatment efficacy. The external transcriptomic plausibility assessment and local parameter-sensitivity analysis were complementary to these three prespecified computational questions. They evaluated the biological coherence of the RGC state orientation and the local robustness of the comparative controller conclusions, respectively, without testing biological treatment efficacy.

## 2. Materials and Methods

### 2.1. Study Design and Scope

This study was designed as a computational neuroscience and software investigation of compact policy learning and regionalized inference in a transcriptomics-informed simulation of optic nerve injury.

The workflow comprised four consecutive components: reconstruction of biologically interpretable injury-state trajectories from public mouse transcriptomic data; development of a spatial, multi-step injury simulator; generation and neural compression of a globally informed model-predictive-control policy; and implementation of the resulting controllers in the BioEdge-RGC interactive application.

The biological component was based exclusively on publicly available single-cell, single-nucleus, and bulk RNA-sequencing datasets obtained from mouse models of optic nerve crush. No new animals, biological specimens, clinical data, or human participants were included. Transcriptomic measurements were used to define the observation variables and temporal reference states of the simulator. They were not used to estimate the causal effects of the simulated interventions.

The intervention-effect vectors, regional coupling coefficients, process-noise distributions, intervention budget, objective-function weights, safety penalties, and failure thresholds were predefined engineering assumptions. Consequently, BioEdge-RGC is described as a data-informed simulation platform rather than an experimentally validated digital twin, biological treatment model, or clinical decision-support system. Conclusions concerning controller performance are restricted to the specified computational environment.

The biological sample, rather than the individual cell or nucleus, was treated as the replication unit for descriptive transcriptomic analyses. Cell-level measurements were aggregated within their original biological samples before temporal statistical comparisons were performed. Individual cells were therefore not treated as independent biological replicates, and no inferential claims were based on cell-level sample sizes.

The replication unit for controller evaluation was the complete simulated episode. Each episode consisted of a spatially heterogeneous initial injury state followed by five sequential decision steps. Controllers evaluated under the same experimental condition were exposed to paired initial states and process-noise realizations wherever applicable. Transcriptomic samples were not divided into neural network training and test sets because the neural policies were trained on simulated state–action episodes rather than directly on individual transcriptomic cells.

The principal computational question was whether a multi-step policy produced by a globally informed controller could be transferred to substantially smaller regional agents operating with incomplete observations and compressed communication. The predefined outcomes included closed-loop objective value, final simulated tissue health, irreversible-failure rate, agreement with the reference controller, policy-retention ratio, model size, inference latency, communication volume, robustness to missing measurements, and performance under out-of-distribution injury severity.

The transcriptomic analyses informed the biological coordinates and temporal organization of the simulated injury state. They did not determine the regional transition equations, intervention-effect vectors, spatial coupling coefficients, objective-function weights, or failure thresholds. Accordingly, the principal outcome of the study was controller performance within the predefined computational environment rather than prediction of a biological treatment response.

**Figure 2:**
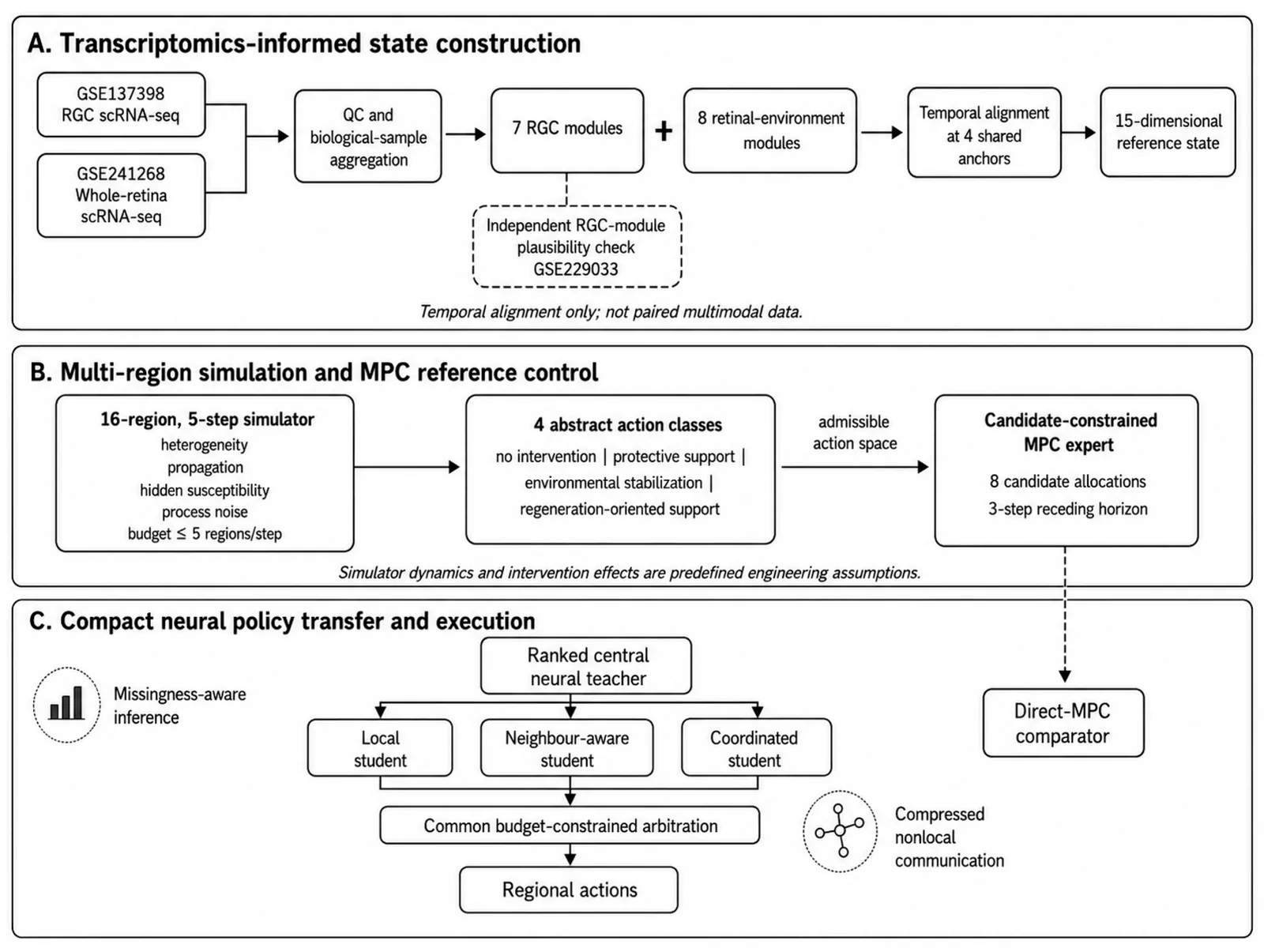
Detailed BioEdge-RGC computational workflow. (**A**) Public optic-nerve-injury transcriptomic datasets were subjected to dataset-specific quality control and biological-sample-level aggregation to construct seven RGC-intrinsic and eight retinal environment modules. The two module sets were temporally aligned at four shared early-injury anchors to generate the 15-dimensional biological reference state. GSE229033 was analyzed independently as an external plausibility assessment of the transferred RGC modules and did not contribute to simulator construction or controller training. (**B**) The reference state was embedded in a heterogeneous 16-region, five-step simulator with four abstract intervention classes and a predefined five-region action budget. A candidate-constrained MPC expert evaluated eight candidate allocations using a three-step receding horizon. Simulator transition dynamics and intervention effects remained predefined engineering assumptions. (**C**) The MPC policy was approximated by a ranked central neural teacher and transferred to local, neighbour-aware, and coordinated regional neural policies operating with different levels of nonlocal information. An architecture-matched Direct-MPC comparator was trained separately from MPC-generated labels rather than from teacher outputs. Final neural-policy actions were subjected to common budget-constrained arbitration before regional action assignment.

The detailed computational workflow is summarized in Figure 2. Public optic-nerve-injury transcriptomic datasets were processed to construct complementary RGC-intrinsic and retinal environment state modules, which were temporally aligned to define the 15-dimensional biological reference state. This state was then embedded in the multi-region simulator used by the candidate-constrained MPC reference controller. The resulting policy was approximated by a ranked central neural teacher and transferred to compact regional policies operating under progressively restricted information and communication conditions. An architecture-matched comparator was trained directly from MPC-generated labels, independently of teacher-based distillation. Figure 2 also distinguishes transcriptomics-derived state construction from the predefined engineering assumptions governing simulator dynamics and intervention effects.

To improve readability, the methods are organized around eight principal methodological components, while detailed simulator, controller, communication, sensitivity-analysis, and reproducibility parameters are provided in the Supplementary Materials.

### 2.2. Transcriptomic Data and Biological-State Construction

#### 2.2.1. Public Transcriptomic Datasets

Four publicly available mouse transcriptomic datasets were considered: GSE137398, GSE241268, GSE248537, and GSE229033 [1,6,16,17,18,19]. GSE137398 provided the longitudinal RGC-intrinsic response after optic nerve crush; GSE241268 represented the broader retinal cellular environment during the early post-injury period; GSE248537 provided an exploratory RGC-enriched transcriptional comparison following Oprm1 overexpression; and GSE229033 provided an independent RGC-specific Dnmt3a perturbation dataset for external transcriptomic plausibility assessment.

Only GSE137398 and GSE241268 contributed variables to the primary 15-dimensional biological state representation. GSE248537 was used solely as descriptive biological context, whereas GSE229033 was used only for independent assessment of the transferred RGC-module orientation. Neither dataset contributed to simulator calibration, policy generation, neural network training, checkpoint selection, or the primary controller benchmark.

##### GSE137398: Longitudinal RGC Response After Optic Nerve Crush

GSE137398 contains single-cell RNA-sequencing data from enriched mouse RGCs collected under uninjured control conditions and at 12 h, 1 day, 2 days, 4 days, 1 week, and 2 weeks after optic nerve crush [1,16]. The original study was designed to characterize molecularly distinct RGC types, their differential resilience to axonal injury, and the transcriptional changes preceding degeneration or survival.

The processed count matrices were aligned with the official RGC metadata. The analysis cohort comprised 76,646 officially annotated optic-nerve-crush RGCs distributed across 32 biological samples and 47 reported clusters or subtypes. Counts were aggregated at the original biological-sample level before construction of the RGC state modules.

The seven longitudinal time points were used to characterize the evolution of RGC identity and function, acute injury and cellular stress, apoptotic pressure, canonical survival signaling, axon-regeneration competence, candidate neuroprotective responses, and interferon-associated inflammatory signaling. The 7- and 14-day observations provided additional degeneration and late-trajectory context, whereas the early common time points were used for temporal integration with the retinal environment dataset.

##### GSE241268: Early Whole-Retina Injury Environment

GSE241268 contains whole-retina single-cell RNA-sequencing data obtained from mice under sham conditions and at 12, 24, and 48 h after optic nerve crush [6,17]. The dataset was generated to examine injury-associated intercellular communication between RGCs and surrounding retinal populations.

Seven biological samples were available: two sham samples, one 12 h sample, two 24 h samples, and two 48 h samples. The downloaded matrices contained 81,318 cells before study-specific quality control. A total of 72,985 cells were retained after conservative filtering.

The retained cells were used to construct eight environmental modules representing Müller-cell reactivity, Müller trophic support, microglial activation, loss of microglial homeostasis, complement activation, cytokine/interferon signaling, vascular activation, and extracellular matrix remodeling.

The single biological sample available at 12 h was retained because this time point was required for early temporal alignment with GSE137398. However, the absence of biological replication at 12 h was explicitly recognized as a limitation. The corresponding environmental state was therefore used as a descriptive trajectory anchor and not as the basis for independent inferential claims.

GSE137398 and GSE241268 originated from different animals, sequencing experiments, and tissue preparations. Their integration was therefore performed only at the level of normalized biological modules aligned to common temporal anchors. The resulting representation was not interpreted as a paired dataset, a direct cell–cell interaction measurement, or evidence of causal communication between the neuronal and environmental compartments.

##### GSE248537: Exploratory Oprm1-Overexpression Context

GSE248537 contains single-nucleus RNA-sequencing data from enriched RGCs collected five days after optic nerve crush, comparing an injury-control condition with RGC-specific overexpression of human *OPRM1* [6,18]. The dataset was generated as part of the experimental investigation that identified the μ-opioid receptor as a candidate neuroprotective factor for RGCs.

One biological sample was available for each experimental arm. Because the dataset lacked biological replication and represented a targeted perturbation at a time point not shared by the two primary trajectory datasets, it was not incorporated into the 15-dimensional state representation, intervention-effect estimation, policy generation, neural network training, or controller comparison.

GSE248537 was retained only as source-study context for the reported Oprm1-associated response and was not subjected to inferential analysis in the present study. No treatment-effect magnitude, statistical efficacy claim, or causal action parameter was derived from this dataset.

##### GSE229033: Independent Dnmt3a-Perturbation Dataset

GSE229033 contains bulk RNA-sequencing profiles from retinal ganglion cells isolated from three control and three RGC-specific Dnmt3a conditional-knockout mice at 2 days after optic nerve crush. The dataset was used solely to assess whether the seven RGC modules defined from GSE137398 showed biologically coherent directional changes in an independent perturbation context. The module definitions were transferred without modification. GSE229033 was excluded from construction of the 15-dimensional state, simulator parameterization, controller training, and evaluation of controller efficacy. The four transcriptomic datasets and their respective roles in BioEdge-RGC are summarized in Table 1.

#### 2.2.2. Quality Control and Construction of the GSE137398 RGC Cohort

The seven processed GSE137398 count matrices corresponded to control, 12 h, 1 day, 2 days, 4 days, 1 week, and 2 weeks after optic nerve crush. The matrices were inspected sequentially to avoid simultaneous loading of the complete gene-by-cell dataset into memory. For each cell, the total number of transcript counts, number of detected genes, mitochondrial transcript count, and mitochondrial transcript fraction were calculated.

A conservative quality-control rule required a positive library size, at least 200 detected genes, and a mitochondrial transcript fraction not exceeding 20%. These criteria were used to identify gross technical failures rather than to redefine the original biological cohort. Sample- and time-point-specific distributions of library size, detected genes, and mitochondrial fraction were inspected before downstream analysis.

The expression matrices contained 106,246 cell barcodes. These barcodes were matched to the official metadata distributed with the original GSE137398 study [1,16]. A total of 76,646 cells had an exact match to an officially annotated optic-nerve-crush RGC. All matched RGCs satisfied the conservative quality-control criteria, and their metadata-derived time labels agreed with the corresponding expression-matrix time points. The remaining 29,600 expression-matrix cells lacked an official RGC annotation and were excluded from the RGC-specific analysis.

No de novo RGC classification or reclustering was performed. The original cluster and subtype assignments were retained to preserve consistency with the source study. The final cohort consisted of 76,646 annotated RGCs from 32 biological samples and 47 reported clusters or subtypes.

Cluster proportions and cell numbers were used only for descriptive quality assessment. They were not interpreted as direct estimates of subtype abundance because the original samples differed in sorting strategy, genetic background, batch, and total number of recovered cells. All subsequent transcriptomic calculations used the original biological sample, rather than the individual cell, as the unit of replication.

#### 2.2.3. Sample-Level RGC Pseudobulk and State Modules

##### Pseudobulk Construction

Raw counts from the retained RGCs were aggregated by gene within each of the 32 original biological samples. The resulting pseudobulk matrix contained 40,790 genes and 32 sample-level profiles. For gene *g* and sample *s*, the pseudobulk count was defined as

(1) $$C_{g,s}=\sum_{i\in s}c_{g,i}$$

where $c_{g,i}$ denotes the count of gene ***g*** in cell ***i***.

Library-size-adjusted counts per million were calculated as

(2) $${CPM}_{g,s}=10^{6}\frac{C_{g,s}}{\sum_{h}C_{h,s}}$$

Genes were retained when they reached CPM≥1 in at least three biological samples. This prespecified exploratory filter retained 16,586 of the 40,790 genes. The filtered values were transformed as

(3) $$L_{g,s}=\log_{2}\left({CPM}_{g,s}+1\right)$$

The raw pseudobulk count matrix was preserved for count-based analyses, whereas the transformed matrix was used for descriptive dimensionality reduction, correlation analysis, and biological state construction.

Principal-component analysis was performed using the 2000 genes with the highest sample-level variance. PCA was used as a global quality and trajectory check rather than as a classifier or inferential endpoint. Sample correlations, genetic background, sorting batch, and injury time were examined jointly to identify potential technical or design-related structure.

Time point, genetic background, and fluorescence-activated sorting strategy were not fully crossed in the original dataset. Consequently, a naive time-only differential-expression model across all 32 samples could confound injury progression with background or sorting effects. The present analysis therefore emphasized transparent module-level trajectories rather than a global time-only differential-expression model.

##### RGC State Modules

Seven prespecified biological modules were used to represent complementary aspects of the RGC response after optic nerve crush:

1. RGC identity and neuronal function;
2. Acute injury and cellular stress;
3. apoptotic pressure;
4. Canonical survival signaling;
5. Axon-regeneration competence;
6. The neuroprotective candidates *Ucn* and *Timp2*;
7. Interferon and inflammatory response.

The modules were defined as transparent sentinel-gene panels rather than comprehensive pathway-enrichment scores. All requested genes were present in the filtered pseudobulk matrix. The complete gene panels are presented in Supplementary Table S1.

Gene overlap between modules was permitted when a sentinel gene had biologically relevant roles in more than one process. Consequently, module scores were not assumed to be statistically independent.

For each retained gene, expression was standardized across the 32 biological samples:

(4) $$Z_{g,s}=\frac{L_{g,s}-{\bar{L}}_{g}}{SD(L_{g})}$$

where ${\bar{L}}_{g}$ and $SD(L_{g})$ denote the mean and sample standard deviation of gene ***g*** across the 32 samples.

The score of module ***k*** in sample ***s*** was calculated as the arithmetic mean of the standardized expression values of its constituent genes:

(5) $$M_{k,s}=\frac{1}{\left|G_{k}\right|}\sum_{g\in G_{k}}Z_{g,s}$$

where $G_{k}$ denotes the gene set assigned to module ***k***.

The seven resulting module scores formed the RGC-intrinsic component of the biological observation vector. Module values were summarized by injury time using the median and interquartile range across biological samples.

Associations between module scores and time after injury were evaluated using Spearman rank correlation. Differences across categorical time points were assessed using the Kruskal–Wallis test. Multiple-testing correction across the seven modules was performed using the Benjamini–Hochberg false-discovery-rate procedure [20]. These analyses were descriptive and were not interpreted as evidence that the module genes causally controlled survival or regeneration.

The *Ucn/Timp2* module was retained separately because these candidates were experimentally evaluated in the original GSE137398 study [1]. Late increases in this module could reflect transcriptional induction, selective survival of resilient RGC types, or both. Accordingly, the module was treated as a state descriptor rather than a treatment-response endpoint. The seven RGC-intrinsic state modules and their constituent sentinel genes are summarized in Table 2.

#### 2.2.4. Retinal Environment State Representation

##### Quality Control and Broad Cellular Assignment

For each of the seven GSE241268 biological samples, the 10X Genomics matrix, barcode, and feature files were read directly. All matrices contained the same 32,285 feature identifiers in the same order.

Cells were retained when they had a positive total count, at least 200 detected genes, and a mitochondrial transcript fraction not exceeding 20%. Of the 81,318 downloaded cells, 72,985 passed these criteria. Retention rates ranged from approximately 88% to 92% across the seven biological samples.

Broad cellular assignments were generated using prespecified marker panels for RGCs, Müller glia, astrocyte-like cells, microglial/myeloid cells, endothelial cells, pericytes, rods, cones, bipolar cells, amacrine cells, and horizontal cells. These assignments were intended to separate major retinal compartments for state construction. They were not presented as the official cell annotations of the original study and were not used to claim the discovery of new retinal subtypes.

Because the principal objective was to represent the tissue environment surrounding injured RGCs, downstream environmental variables were derived from Müller glia, microglial/myeloid cells, endothelial cells, and the pooled non-neuronal retinal compartment.

##### Cell-Type-Level Pseudobulk Scores

Within each biological sample, counts were aggregated separately for the relevant broad cellular compartments. Cell-type pseudobulk counts were converted to CPM and transformed as $\log_{2}\left(CPM+1\right)$, following the same general normalization principle used for the RGC pseudobulk analysis.

Eight environmental state variables were defined:

- Müller reactivity;
- Müller trophic support;
- Microglial activation;
- Loss of microglial homeostasis;
- Microglial complement activity;
- Microglial cytokine/interferon signaling;
- Vascular activation;
- Extracellular matrix remodeling.

Müller reactivity and trophic-support scores were calculated from the Müller-glia pseudobulk profiles. Microglial activation, homeostatic state, complement, and cytokine/interferon scores were calculated from the microglial/myeloid profiles. Vascular activation was calculated from endothelial profiles. Extracellular matrix remodeling was calculated from the pooled non-neuronal environment.

Microglial homeostatic loss was represented as the negative of the homeostatic-gene score, so that larger values consistently indicated a greater departure from the homeostatic state:

(6) $$M_{homeostaticloss,s}=-M_{homeostasis,s}$$

For each module, the transformed expression values of the prespecified genes were averaged within the relevant sample and cellular compartment. The resulting module values were then standardized across the seven biological samples. The full marker and module definitions are provided in Supplementary Table S1. The eight retinal environment modules, corresponding cellular compartments, and principal genes are summarized in Table 3.

The biological sample was the unit of analysis. Cell-level measurements were not treated as independent replicates, and no cell-level statistical significance was used to support environmental trajectory claims. The single 12 h biological sample was retained as a descriptive temporal anchor but did not permit an estimate of between-sample variability at that time point.

#### 2.2.5. Temporal Fusion into the 15-Dimensional Observation State

The RGC and retinal environment datasets were generated from different animals, cellular preparations, sequencing experiments, and sample designs. No direct pairing of biological samples was therefore possible. Fusion was restricted to four shared temporal anchors:

- Control in GSE137398 with sham in GSE241268;
- 12 h with 12 h;
- 1 day with 24 h;
- 2 days with 48 h.

For each RGC module, the median score across the biological samples available at the corresponding time point was used as the neuronal temporal anchor because several biological samples were available at most time points and a robust summary was preferred. For each environmental module, the arithmetic mean of the available biological samples was used as the environmental anchor. The environmental time points contained only one or two samples; for two observations, the mean and conventional sample median correspond to the same midpoint, whereas both reduce to the single available value at 12 h. Interquartile ranges for the RGC modules and sample ranges for the environmental modules were retained as descriptive uncertainty summaries.

Each temporal series was expressed relative to its own baseline:

(7) $$∆x_{k,t}=x_{k,t}-x_{k,0}$$

where $x_{k,0}$ denotes the control or sham value of module ***k***.

To place modules with different numerical ranges on a common scale, each baseline-referenced trajectory was divided by its largest absolute early-injury deviation:

(8) $${\tilde{x}}_{k,t}=\frac{∆x_{k,t}}{\underset{u\in \left\{12h,1d,2d\right\}}{\max}\left|∆x_{k,u}\right|}$$

The control value was therefore zero, and the largest absolute deviation of each module across the three post-injury anchors was mapped to either +1 or −1. This transformation preserved the direction and relative temporal evolution of each module while preventing modules with larger raw numerical ranges from dominating the simulator.

The fused biological state at time ***t*** was defined as

(9) $$x_{t}=\left[x_{t}^{RGC},x_{t}^{ENV}\right]\in R^{15}$$

where

$$x_{t}^{RGC}\in R^{7}$$

contained the seven RGC-intrinsic modules and

$$x_{t}^{ENV}\in R^{8}$$

contained the eight retinal environment modules.

The resulting four-point trajectory served as the transcriptomics-informed temporal reference trajectory from which initial simulated regional states were generated. The fusion was temporal and descriptive. It was not interpreted as a paired multi-omic dataset, a reconstructed cell–cell interaction network, or evidence that changes observed in one dataset causally produced changes in the other.

Only the 15 module values were supplied as biological state variables. Dataset identity, sample identifiers, original RGC subtype labels, and individual-cell expression profiles were not provided directly to the controllers. This separation reduced the risk that model performance would depend on sample-specific technical information rather than on the intended injury-state representation.

##### Independent Transcriptomic Plausibility Assessment

To assess whether the prespecified RGC modules showed biologically coherent behavior in an external perturbation dataset, GSE229033 was analyzed independently of the datasets used to construct the BioEdge-RGC temporal reference trajectory. GSE229033 contains bulk RNA-sequencing profiles from retinal ganglion cells isolated from three control and three RGC-specific Dnmt3a conditional-knockout mice at 2 days after optic nerve crush.

The seven RGC module definitions reported in Supplementary Table S1 were transferred without modification. Raw sample-level counts were converted to counts per million, transformed as log_2_(CPM + 1), standardized gene-wise across the six samples, and averaged across the prespecified genes of each module. All genes included in the seven modules were represented in the external dataset.

A favorable Dnmt3a-cKO response was defined before evaluation as increased RGC identity/function, canonical survival signaling, axon-regeneration competence, and candidate-neuroprotective response together with decreased acute injury/stress, apoptotic pressure, and interferon/inflammatory response. Sample-level module differences, Hedges’ g effect sizes, and stratified-bootstrap 95% intervals based on 5000 resamples were reported. An exploratory equal-weight oriented composite was calculated by multiplying each module score by its prespecified favorable orientation and averaging the seven oriented scores. Because the dataset contained only three biological samples per group, interpretation emphasized effect direction and magnitude rather than null-hypothesis significance testing. This analysis evaluated transcriptomic plausibility of the RGC state representation and did not validate the simulator transition equations, intervention-effect vectors, MPC decisions, or controller efficacy.

### 2.3. Multi-Region Optic Nerve Injury Simulator

#### 2.3.1. Spatial Organization and Episode Structure

The 15-dimensional biological state was embedded in a spatial simulator comprising 16 interconnected tissue regions. The regions were arranged in a circular topology so that each region had two immediate neighbours. This simplified geometry was selected to represent local propagation and regional heterogeneity without claiming anatomical reconstruction of the retina or optic nerve.

Each simulation episode contained five sequential decision steps. At each step, the controller observed the available regional measurements, assigned one action class to each region, and selected no more than five regions for active intervention. The five-region limit represented a predefined intervention budget rather than a biologically measured treatment capacity.

For region *i* at decision step *t*, the latent state was denoted by

(10) $$x_{i,t}\in R^{15}$$

where the first seven components represented RGC-intrinsic state and the remaining eight components represented the retinal environment.

The complete latent tissue state was

(11) $$X_{t}=\left[x_{1,t},x_{2,t},\ldots ,x_{16,t}\right]$$

The latent state represented the internal simulated tissue condition. Controllers did not necessarily receive the complete latent state because sensor missingness and architecture-specific information restrictions were applied separately to the observations.

#### 2.3.2. Initialization of Regional States

Initial regional states were sampled around the biological temporal anchors obtained from the fused GSE137398–GSE241268 trajectory. Episode-level severity, local susceptibility, spatial direction, environmental burden, and stochastic variability were sampled independently using predefined distributions.

The initial state of region ***i*** was represented as

(12) $$x_{i,0}=x_{anchor}+\delta_{i}+\eta_{i}$$

where $x_{anchor}$ was a selected biological reference state, $\delta_{i}$ represented structured regional heterogeneity, and $\eta_{i}$ represented stochastic variation.

Two broad spatial patterns were simulated. Directional episodes contained a primary injury wave that propagated preferentially around the regional ring. Multifocal episodes contained several partially independent high-hazard regions. These patterns were intended to create a nontrivial allocation problem rather than to reproduce the physical geometry of optic nerve crush.

Each region also received a hidden susceptibility parameter that influenced the accumulation of injury. Susceptibility affected the latent transition dynamics but was not directly available as a controller input.

#### 2.3.3. Abstract Intervention Classes

Four action classes were defined:

(13) $$a_{i,t}\in \left\{0,1,2,3\right\}$$

where

- $a_{i,t}=0$ represented no intervention;
- $a_{i,t}=1$ represented acute protective support;
- $a_{i,t}=2$ represented environmental or inflammatory stabilization;
- $a_{i,t}=3$ represented regeneration-oriented support.

These actions were abstract engineering classes. They did not represent specific drugs, gene therapies, stimulation protocols, or experimentally measured treatment effects.

Each active action was associated with a predefined signed effect vector acting on selected components of the 15-dimensional state. The complete vectors and their numerical coefficients are reported in Supplementary Table S2. The values were fixed before the final V3.2 benchmark and were not adjusted after examination of the test results.

The three active intervention classes were designed to create temporally competing objectives. Protective and environmental stabilization actions were favoured during high acute hazard, whereas regeneration-oriented support was potentially beneficial only after sufficient stabilization. Premature application of regeneration-oriented support during severe acute stress incurred an explicit adverse penalty.

#### 2.3.4. Regional Transition Dynamics

The implemented regional transition rule was

(14) $$x_{i,t+1}={clip}_{\left[-2.0,2.2\right]}\left[x_{i,t}+\chi_{i}{B(a}_{i,t})+d_{i,t}+c_{i,t}+\varepsilon_{i,t}\right]$$

where $\chi_{i}$ was the hidden regional susceptibility, ${B(a}_{i,t})$ was the predefined 15-dimensional action-effect vector, $c_{i,t}$ represented neighbour-mediated propagation, $d_{i,t}$ collected deterministic injury, timing, and late-step drift terms, and $\varepsilon_{i,t}$ represented process noise. Susceptibility multiplied the intervention-effect vector but was not supplied to the controller.

Regional hazard was defined as the arithmetic mean of state dimensions 2, 3, 7, 10, 11, 12, and 13. Untreated hazard was defined as

$$u_{i,t}=\max\left(0,h_{i,t}-0.10\right)I\left(a_{i,t}=0\right)$$

The direction-dependent incoming propagation signal was

(15) $$p_{i,t}=0.24\left\{\begin{array}{cc} u_{i-1,t}, & clockwise\\ u_{i+1,t,} & counter-clockwise\\ 0.55\left(u_{i-1,t}+u_{i+1,t}\right), & multifocal \end{array}\right.$$

with circular indexing of the 16 regions. The propagation signal modified acute injury stress, apoptotic pressure, interferon/inflammatory response, microglial activation, microglial complement, and microglial cytokine/interferon dimensions using multipliers of 1.00, 0.85, 0.40, 0.50, 1.05, and 1.00, respectively.

After intervention and propagation, severe hazard was defined as $\max\left(0,h_{i,t+1}-0.55\right)$. This term reduced RGC identity/function, canonical survival signaling, and axon-regeneration competence using coefficients of 0.13, 0.05, and 0.08, respectively. Regeneration-oriented support applied during decision steps 0 or 1 incurred an additional penalty when pre-action hazard exceeded 0.30, increasing acute injury stress, apoptotic pressure, and microglial cytokine/interferon signaling by 0.16, 0.14, and 0.10 times the premature-action signal, respectively.

At decision steps 2–4, deterministic drift decreased acute injury stress by 0.025 and apoptotic pressure by 0.018 and increased Müller trophic support by 0.020 per step. Process noise was then added, and each state component was clipped to the interval [−2.0, 2.2] for numerical stability.

The complete initialization distributions, transition coefficients, action vectors, clipping ranges, noise parameters, susceptibility distributions, and failure thresholds are provided in Supplementary Table S2.

#### 2.3.5. Objective Function

Controller performance was assessed using a common five-step objective combining final simulated tissue health, mean health across the complete trajectory, intervention burden, and irreversible failure. Premature regeneration-oriented support was penalized through the transition dynamics described in Section 2.3.4 rather than through a separate objective-function term.

For one episode, the implemented objective was

(16) $$J=0.60H_{T}+0.40\bar{H}-0.025A-0.12F$$

where $H_{T}$ was the mean final simulated health across the 16 regions, $\bar{H}$ was mean simulated health across the initial state and all five post-decision states and across all regions, ***A*** was the fraction of region–step decisions assigned an active action, and ***F*** was the binary irreversible-failure indicator. Regional health was calculated as one minus the weighted state-loss function defined by the state orientations, weights, and time-dependent repair-support thresholds reported in Supplementary Table S2.

Irreversible failure was recorded when either the minimum final RGC identity/function value across regions was below −1.25 or the maximum final regional hazard was above 1.05. All controllers were evaluated using this same objective. The objective was an engineering benchmark and was not interpreted as a validated biological recovery score.

### 2.4. Reference and Neural Control Policies

#### 2.4.1. Candidate-Constrained Model Predictive Control Expert

The reference controller was a candidate-constrained, receding-horizon model predictive control expert [8]. The term expert is used instead of oracle because the controller did not evaluate every mathematically possible future action sequence and did not have access to future process-noise realizations.

At each decision step, the MPC expert received the complete current latent state of all 16 regions. A fixed set of eight candidate action allocations was constructed from the current regional risks, hazards, repair states, incoming-hazard gradients, and action-timing rules. One candidate assigned no intervention to all regions. Each of the remaining seven candidates assigned an active action to exactly five regions, thereby satisfying the five-region intervention budget.

For a current candidate allocation *u*, the first projected state was

(17) $${\hat{X}}_{t+1}^{\left(u\right)}=f\left(X_{t},u\right)$$

where ***f*** represented the deterministic transition model without access to future process-noise realizations. When the three-step horizon extended beyond the current decision, subsequent projected transitions used the predefined greedy mixed-action candidate generated from the corresponding projected state. Terms extending beyond the end of the five-step episode were omitted.

The implemented candidate score was

(18) $$Q_{t}\left(u\right)=0.35{\hat{H}}_{t+1}^{\left(u\right)}+0.35{\hat{H}}_{t+2}^{\left(u\right)}+0.30{\hat{H}}_{t+3}^{\left(u\right)}-0.015A(u)$$

where ${\hat{H}}_{t+h}^{\left(u\right)}$ was the mean projected regional health at horizon step *h*, and A(u) was the fraction of regions assigned an active action in the current candidate. The allocation with the highest projected value was selected:

(19) $$u_{t}^{*}=\arg\underset{u\in U_{t}}{\max}\;Q_{t}\left(u\right)$$

Only the first action allocation was applied. The state was then updated, new observations were generated, and planning was repeated at the next decision step. The MPC expert was used to generate reference action labels, regional-priority targets, and complete closed-loop comparator trajectories. It was not assumed to be globally optimal.

#### 2.4.2. Ranked Central Neural Teacher

##### Teacher Inputs

The central teacher received the complete state of all 16 regions together with the current decision step and remaining normalized intervention budget. The input at step ***t*** was therefore

(20) $$T_{t}^{teacher}=\left\{X_{t},\tau_{t},b_{t}\right\}$$

where $\tau_{t}$ represented normalized decision time and $b_{t}$ represented the remaining budget.

Because regional indices were not intended to carry intrinsic biological meaning, the teacher was designed to be equivariant to permutation of the 16 regional inputs. Each region was initially processed using the same local encoder:

(21) $$h_{i,t}=\phi (x_{i,t}{,\tau }_{t},b_{t})$$

Global context was constructed by combining mean and maximum pooling:

(22) $$g_{t}=\left[\frac{1}{16}\sum_{i=1}^{16}h_{i,t}\underset{i}{\max}\;h_{i,t}\right]$$

The regional embedding and global context were concatenated before prediction:

(23) $$z_{i,t}=\left[h_{i,t},g_{t}\right]$$

This design followed the general principle of permutation-invariant set processing [21].

##### Teacher Outputs

Two prediction heads were used. The action head produced a probability distribution over the four action classes:

(24) $$p_{i,t}^{teacher}=softmax\left(W_{a}z_{i,t}+b_{a}\right)$$

The priority head produced a continuous regional priority value:

(25) $$q_{i,t}^{teacher}=W_{q}z_{i,t}+b_{q}$$

The four-class probabilities were retained for training and agreement analysis. For final budget-constrained neural execution, the candidate active class for each region was defined as the highest-probability class among action classes 1–3. The five regions with the highest priority scores were assigned their candidate active classes, and all remaining regions were assigned no intervention.

Teacher training combined action classification, prediction of MPC-selected regions, and pairwise regional ranking. The teacher therefore learned both the preferred action class and the relative allocation priority under the global intervention budget.

#### 2.4.3. Missingness-Aware Regional Neural Policies

Three compact student architectures were developed: local, neighbour-aware, and coordinated.

##### Sensor Missingness and Imputation

Sensor failure was applied only to controller observations. The underlying latent simulated tissue state was not altered.

For biological variable ***k*** in region ***i***, the observation mask was

(26) $$m_{i,t,k}\in \left\{0,1\right\}$$

where $m_{i,t,k}=1$ indicated that the variable was available.

The observed value was

(27) $$o_{i,t,k}=m_{i,t,k}x_{i,t,k}+\left(1-m_{i,t,k}\right){\tilde{x}}_{k}$$

where ${\tilde{x}}_{k}$ was the median value of variable ***k*** estimated from the training data.

The 15-element availability mask was supplied explicitly to the student together with the imputed state. This allowed the model to distinguish an observed median-like value from an imputed missing value.

During student training, between 0% and 30% of biological measurements were randomly masked. Progressive missingness robustness was evaluated on the fixed severe-injury OOD episode set at predefined missingness levels of 0%, 10%, 25%, and 40%.

##### Local Student

The local student received only the imputed state and availability mask of region *i*, together with decision time and remaining budget:

(28) $$T_{i,t}^{local}=\left[o_{i,t},m_{i,t},\tau_{t},b_{t}\right]$$

The local student did not receive the states, messages, or predictions of other regions during neural inference. Its regional action probabilities and allocation-priority scores were subsequently processed by the common budget-arbitration mechanism described in Section Budget-Constrained Arbitration and Degree of Decentralization.

##### Neighbour-Aware Student

The neighbour-aware student additionally received compressed summaries of the two adjacent regions. Six continuous neighbour-gradient features were used to represent local differences in injury hazard, environmental burden, repair state, and recent state evolution. Four additional indicators described neighbour-message availability.

Its input was

(29) $$T_{i,t}^{neighbour}=\left[T_{i,t}^{local},n_{i,t},m_{i,t}^{neighbour}\right]$$

The student did not receive complete neighbouring state vectors.

##### Coordinated Student

The coordinated student received the local and neighbour-aware inputs together with a compact global broadcast. The global broadcast contained four tissue-level hazard and allocation summaries and four corresponding availability indicators:

(30) $$T_{i,t}^{coord}=\left[T_{i,t}^{neighbour},g_{t}^{compact},m_{t}^{global}\right]$$

The coordinated student therefore received limited tissue-level context but did not receive the complete 16-region state. The compact global broadcast was calculated by the coordinating layer from predefined aggregate hazard and allocation summaries and was transmitted identically to all regional models.

All regional policies used separate action-class and allocation-priority heads. Their outputs were subsequently passed to the common budget-arbitration mechanism, which selected the five highest-priority regions for active intervention at each neural-policy decision step.

##### Budget-Constrained Arbitration and Degree of Decentralization

Each regional neural model generated a four-class action distribution and a scalar allocation-priority score for its corresponding tissue region. Final neural-policy execution used a common deterministic budget-constrained arbitration procedure.

For every region, the candidate active class was defined as the highest-probability class among the three active classes. Regions were then ranked solely by their allocation-priority scores. The five highest-ranked regions were assigned their corresponding candidate active classes, whereas the remaining 11 regions were assigned no intervention. Consequently, the central teacher and all regional neural policies used exactly five active regions at each decision step. The no-intervention probability remained part of the four-class training output and agreement analysis but did not determine the final budgeted neural allocation. The MPC comparator was handled separately and retained an explicit all-no-intervention candidate within its eight-candidate set.

BioEdge-RGC therefore implemented regionalized neural inference followed by lightweight centralized budget arbitration. It should not be interpreted as a fully autonomous or fully decentralized multi-agent control system. The local architecture used only regional observations during neural inference, the neighbour-aware architecture additionally used compact information from adjacent regions, and the coordinated architecture also received a compact tissue-level broadcast.

#### 2.4.4. Training Objectives: Hybrid Knowledge Distillation and Direct MPC Supervision

Two training strategies were evaluated for transferring the MPC-generated policy to compact regional neural models. The first used a hybrid knowledge-distillation objective combining hard supervision from the MPC reference controller with soft action probabilities and regional-priority targets generated by the central neural teacher [11]. The second used direct supervision from the MPC-generated labels without teacher-probability or teacher-priority terms. This architecture-matched comparison was included to determine whether teacher-based soft supervision provided an advantage beyond direct behavioural cloning of the same MPC policy.

For the hybrid-distilled models, the total training loss was

(31) $$L=\lambda_{CE}L_{CE}+\lambda_{KD}L_{KD}+\lambda_{active}L_{active}+\lambda_{rank}L_{rank}+\lambda_{priority}L_{priority}$$

where the loss weights were fixed before final model evaluation.

The numerical loss weights, distillation temperature, ranking margin, optimizer settings, batch size, and complete checkpoint-selection history were not retained in the final V3.2 artifacts. Equations (31)–(35) therefore document the structure of the training objective, whereas reproducibility of the reported benchmark is based on the locked checkpoints and evaluation code supplied in Supplementary File S1. Exact retraining from random initialization is outside the reproducibility scope of the retained package.

The hard-label action loss was

(32) $$L_{CE}=-\sum_{i,c}y_{i,c}^{MPC}\log p_{i,c}^{student}$$

The distillation component minimized the divergence between temperature-scaled teacher and student action probabilities:

(33) $$L_{KD}=T^{2}KL\left(p_{T}^{teacher}|p_{T}^{student}\right)$$

where ***T*** was the distillation temperature.

The active-region loss was a binary cross-entropy term indicating whether each region was selected by the MPC expert. Pairwise ranking required MPC-selected regions to receive higher priority than untreated regions:

(34) $$L_{rank}=\frac{1}{\left|P\right|}\sum_{(i,j)\in P}\max\left[0,\mu -q_{i}^{student}+q_{j}^{student}\right]$$

where ***i*** was a selected region, ***j*** was an untreated region, and μ was the ranking margin.

Teacher-priority regression was represented by

(35) $$L_{priority}=\frac{1}{16}\sum_{i=1}^{16}{\left(q_{i}^{student}-q_{i}^{teacher}\right)}^{2}$$

An additional coordinated student with identical architecture was trained directly from MPC labels without the teacher-probability and teacher-priority terms. This direct-MPC model served as a behavioural cloning comparator. It prevented improved performance from being attributed to distillation when the same architecture could learn equally well from hard expert labels alone.

### 2.5. Communication and Software Implementation

#### 2.5.1. INT8 Communication Codec

Only the continuous neighbour and global summary messages were passed through the communication codec. Each continuous value ***v*** was first clipped to the fixed interval [−2, 2] and then converted using symmetric signed-integer quantization [22]:

(36) $$v_{c}=clip\left(v,-2,2\right),\;\;q=clip\left[round\left(\frac{127v_{c}}{2}\right),-127,127\right]$$

(37) $$\hat{v}=\frac{2q}{127}$$

At the receiving node, the reconstructed value was

Availability summaries were transmitted separately and were not passed through the continuous-message quantizer. The clipping interval and scale were fixed by the implementation and were not estimated from the test data.

Modeled communication volume was reported in software bytes per complete five-step episode. The retained V3.2 output verifies aggregate totals of 5520 bytes for centralized teacher execution, 400 bytes for local execution, 1000 bytes for neighbour-aware execution, and 1080 bytes for coordinated execution. The component-level byte ledger used to construct these totals was not retained. These values represent an abstract digital communication model and are not measurements of molecular signaling cost, wireless power consumption, implant energy, or biological diffusion.

Separately, the final coordinated student was converted after training using dynamic INT8 model quantization to evaluate checkpoint-size and latency effects. This model conversion did not alter the communication codec or the modeled number of transmitted bytes per episode.

#### 2.5.2. BioEdge-RGC Interactive Application

BioEdge-RGC was implemented as a local browser-based research application linked to the locked policy checkpoints. The application was developed in Python 3.12 and used a Gradio 6.5.1 interface for interactive execution.

The user can define:

- The reproducibility seed;
- Standard or out-of-distribution injury severity;
- Directional or multifocal propagation;
- Sensor-missingness level;
- Controller type;
- Intervention budget.

The available policies include:

- No intervention;
- Fixed protocol;
- Candidate-constrained MPC expert;
- Central neural teacher;
- Local student;
- Neighbour-aware student;
- Coordinated student;
- Direct-MPC coordinated student;
- INT8-coded coordinated student.

For each five-step episode, the interface displays the 16-region state map, regional hazard, sensor availability, selected action class, remaining intervention budget, mean RGC-identity trajectory, environmental-hazard trajectory, final simulated health, objective value, and irreversible-failure status.

The complete regional action schedule can be exported as a comma-separated values file. Latent states, incomplete observations, availability masks, controller probabilities, regional priorities, selected actions, and episode-level outcomes can be exported in JSON format. The current implementation does not provide a clinical interoperability format. In a future experimentally and clinically validated implementation, structured observations, simulated interventions, device states, and outcomes could be mapped to standards such as HL7 FHIR, whereas DICOM-compatible exchange could be considered if imaging-derived inputs or outputs were incorporated. Such interoperability could facilitate integration with medical-simulation environments, patient-specific phantoms, or research-oriented digital-twin platforms. These possibilities represent prospective engineering extensions only; the present CSV and JSON exports are intended for research reproducibility and software inspection and should not be interpreted as clinical-data exchange functionality.

The application was designed for reproducible software inspection and educational demonstration. It is not a medical device, clinical decision-support system, or validated model of therapeutic response.

### 2.6. Training, Validation, and Test Protocol

Training, validation, held-out test, and out-of-distribution (OOD) episode sets were generated using mutually exclusive random seeds. Neural models were trained exclusively on simulated state–action examples produced by the locked simulator and the candidate-constrained MPC reference controller. Neither the held-out test episodes nor the OOD episodes were used for model fitting, hyperparameter selection, checkpoint selection, or adjustment of the simulator.

The final held-out test set comprised 550 simulated episodes, and the severe out-of-distribution evaluation set comprised 550 episodes. The progressive-missingness analysis reused the same 550 latent OOD episodes at each masking level, with only the observation-availability masks being changed.

Hyperparameters and final model checkpoints were selected using the validation set. The simulator coefficients, intervention-effect vectors, objective-function weights, failure thresholds, communication-accounting rules, evaluation outcomes, and primary success criteria were frozen before execution of the final V3.2 benchmark.

The primary held-out evaluation was performed with 10% observation missingness. This missingness level was applied only to the observations provided to the controllers; the corresponding latent simulated tissue states were not modified.

Progressive missingness robustness was evaluated separately on the fixed severe-injury OOD episode set at observation-missingness levels of 0%, 10%, 25%, and 40%. The same latent initial states, regional susceptibility parameters, propagation patterns, and process-noise realizations were preserved across all four missingness conditions. Only the availability masks and the resulting controller observations were changed. Consequently, the absolute objective and failure-rate values obtained in the progressive-missingness analysis are not directly comparable with those of the primary held-out benchmark.

OOD episodes contained higher initial injury severity, stronger regional propagation, increased hidden susceptibility, and broader initial spatial heterogeneity than the training distribution. All OOD conditions remained within the same predefined simulator family. The OOD analysis therefore evaluated within-simulator extrapolation under more severe and heterogeneous conditions and did not represent biological generalization to another animal model, species, injury mechanism, or experimental platform.

### 2.7. Evaluation Outcomes and Robustness Analysis

#### 2.7.1. Evaluation Outcomes and Statistical Analysis

The primary evaluation outcome was the mean five-step episode objective. Secondary outcomes included final simulated tissue health, irreversible-failure rate, regional action agreement with the MPC reference controller, absolute objective difference from the reference policy, performance-retention ratio, model parameter count, serialized checkpoint size, modeled communication volume per episode, inference latency, robustness to missing observations, and performance under severe out-of-distribution injury.

Performance retention of model ***A*** relative to reference model ***B*** was calculated as

(38) $$Retention\left(A,B\right)=100\frac{J_{A}}{J_{B}}$$

Because this ratio depends on the numerical origin and scaling of the simulated objective, retention was interpreted together with the absolute objective difference

(39) $$∆J_{A-B}=J_{A}-J_{B}$$

where negative values indicate that model ***A*** achieved a lower mean objective than reference model ***B***.

Relative failure reduction was calculated as

(40) $$Failure\;Reduction=100\frac{F_{reference}-F_{model}}{F_{reference}}$$

where ***F*** denotes the episode-level irreversible-failure rate. Absolute differences in failure rate were additionally reported in percentage points.

Communication saving relative to centralized execution was calculated as

(41) $$Communication\;Saving=100\left(1-\frac{C_{edge}}{C_{central}}\right)$$

where ***C*** denotes the modeled software communication volume per complete five-step episode.

Controllers were evaluated on paired episodes generated from identical initial latent states and process-noise realizations. Controller differences were therefore calculated episode by episode before aggregation. Episode outcomes were summarized using means and empirical 2.5th–97.5th percentiles across simulated episodes. These empirical ranges describe variation among episodes within the specified simulator and should not be interpreted as confidence intervals for biological treatment effects, animal populations, or clinical outcomes.

The prespecified policy-approximation criteria were objective retention of at least 97% for the central neural teacher relative to the MPC reference controller and at least 96% for the coordinated student relative to the central teacher. The prespecified coordinated-versus-local practical thresholds were a mean-objective difference of at least +0.008 and a relative failure reduction of at least 15%. These thresholds were treated as practical decision criteria rather than as null-hypothesis significance tests.

Neural network parameter counts and serialized checkpoint sizes were recorded directly from the final models. Inference latency was measured on the development workstation after model warm-up using repeated execution of one complete 16-region decision step. These latency measurements characterize the tested software and hardware configuration and do not represent implant-level, molecular, or biological execution speed.

All absolute differences, retention ratios, relative reductions, and other derived quantities were calculated from unrounded episode-level values. Mean objectives and other summary outcomes are displayed in the text and tables after rounding for presentation.

#### 2.7.2. Local Parameter-Sensitivity Analysis

A one-factor-at-a-time sensitivity analysis was performed to determine whether the comparative controller findings depended narrowly on the baseline simulator coefficients. A separate fixed cohort of 550 standard-severity episodes was generated using fixed reproducibility seeds and 10% observation missingness. The same initial states, spatial patterns, hidden regional susceptibilities, observation masks, and underlying process-noise realizations were used across all sensitivity conditions. The baseline-parameter condition used the locked V3.2 parameter values but a newly generated sensitivity cohort and was not intended to reproduce numerically the original held-out benchmark reported in the primary held-out analysis.

Four parameter families were varied independently. The three active-action effect vectors were multiplied by 0.90 or 1.10. The neighbour-propagation coefficient was multiplied by 0.90 or 1.10, changing its baseline value of 0.24 to 0.216 or 0.264. The stored standard-condition process-noise realizations were multiplied by 0.90 or 1.10. Finally, the irreversible-failure definition was modified in two directions: the stricter condition used an RGC-identity threshold of −1.125 and a maximum-hazard threshold of 0.945, whereas the more permissive condition used corresponding thresholds of −1.375 and 1.155. All remaining simulator parameters were held at their locked V3.2 values.

Six controllers were evaluated: the MPC expert, central neural teacher, local ranked student, neighbour-aware ranked student, coordinated ranked student, and architecture-matched coordinated direct-MPC student. All neural checkpoints remained fixed and were not retrained. For the action-effect and propagation perturbations, the MPC expert planned using the corresponding perturbed deterministic transition model. Process-noise scaling and changes in the failure definition affected episode execution or evaluation but did not alter the deterministic MPC planning model.

Mean episode objective, empirical 2.5th–97.5th percentile range, mean final simulated health, irreversible-failure rate, objective difference from the scenario-specific MPC expert, performance retention, coordinated-minus-local objective difference, and controller ranking were evaluated. This analysis assessed local robustness of the locked policies to moderate simulator misspecification. It was not interpreted as biological validation or as a global sensitivity analysis.

### 2.8. Reproducibility and Software Availability

All retained V3.2 simulation, model-predictive-control, locked neural-inference, evaluation-verification, visualization, and application code was implemented in Python. Supplementary File S1 contains the retained simulator and MPC implementation, locked teacher and regional-policy checkpoints, the 15-dimensional temporal reference anchors, primary aggregate outputs, the interactive BioEdge-RGC application, verification scripts, an integrity manifest, and execution instructions. Supplementary File S2 contains the Google Colab notebook, standalone Python script, fixed seeds, configuration files, run-level outputs, controller summaries, and structural-comparison results for the local parameter-sensitivity analysis. Supplementary File S3 contains the analysis code, sample-level module scores, gene-coverage audit, effect-size tables, bootstrap summaries, and figure-generation outputs for the independent GSE229033 analysis. Supplementary Tables S1–S4 and Supplementary Figure S1 provide the corresponding definitions and results.

The original V3.2 model-training script and its optimizer and loss hyperparameters were not retained; consequently, the supplied package supports deterministic simulation, inference, application execution, and verification of the retained aggregate results, but not exact retraining from random initialization. The raw CPU-latency benchmark script and the component-level communication byte ledger were also not retained.

The source transcriptomic data are publicly available through the NCBI Gene Expression Omnibus under accessions GSE137398, GSE241268, GSE248537 and GSE229033. No patient-level or newly generated animal data are included.

## 3. Results

The Results are organized to follow the progression from biological-state construction to controller evaluation. First, the RGC-intrinsic and retinal environment transcriptomic trajectories are characterized separately, followed by their temporal integration into the 15-dimensional reference state and an independent assessment of the prespecified RGC-module orientation. We then evaluate the candidate-constrained MPC expert and determine how closely the central neural teacher and compact regional policies reproduce its closed-loop behavior. Subsequent analyses examine the contribution of nonlocal information, the trade-off between performance and modeled communication or model complexity, and robustness to progressive sensor missingness and out-of-distribution injury conditions. Local parameter-sensitivity analyses are then used to determine which comparative controller conclusions remain stable under moderate simulator perturbations. Finally, the trained controllers are demonstrated within the reproducible BioEdge-RGC interactive research application.

### 3.1. The RGC Transcriptomic Trajectory Captured Progressive Loss of Neuronal Identity Together with Injury and Regeneration-Associated Responses

Alignment of the seven GSE137398 expression matrices with the official metadata yielded a final cohort of 76,646 annotated RGCs from 32 biological samples. The retained cells represented 47 reported RGC clusters or subtypes and covered control, 12 h, 1 day, 2 days, 4 days, 1 week, and 2 weeks after optic nerve crush [1,16].

Aggregation at the biological-sample level produced a pseudobulk matrix containing 40,790 genes. Following the prespecified expression filter, 16,586 genes were retained for downstream analysis. Principal-component analysis of the 2000 most variable genes showed a strong organization of the samples along the post-injury trajectory. The first principal component explained 56.6% of the total variance and was strongly correlated with time after injury (Spearman ρ=0.937, *p* = 3.19 × 10^−15^).

The seven RGC state modules showed distinct but coordinated temporal patterns. The RGC identity/function module decreased strongly with time after injury (Spearman ρ=−0.907, FDR-adjusted *p* = 5.68 × 10^−12^). This decline was consistent with the progressive loss of the transcriptional programs associated with mature RGC identity and neuronal function.

In contrast, the acute injury/stress module increased over time (ρ=0.667, FDR-adjusted *p* = 3.51 × 10^−5^). Apoptotic pressure showed an even stronger positive temporal association (ρ=0.852, FDR-adjusted *p* = 1.48 × 10^−9^), indicating that the loss of neuronal identity was accompanied by progressive activation of cell-death-associated programs.

The axon-regeneration-competence module also increased with time (ρ=0.861, FDR-adjusted *p* = 9.00 × 10^−10^). Similarly, the *Ucn/Timp2* candidate-neuroprotection module increased across the injury trajectory (ρ=0.840, FDR-adjusted *p* = 3.29 × 10^−9^). These increases did not indicate successful regeneration at the tissue level. Instead, they showed that transcriptional programs associated with attempted repair and candidate resilience emerged concurrently with degeneration.

The interferon/inflammatory-response module also increased significantly with time (ρ=0.693, FDR-adjusted *p* = 1.54 × 10^−5^). By contrast, canonical survival signaling did not follow a significant monotonic trajectory (ρ=−0.137, FDR-adjusted *p* = 0.455). Thus, survival-associated transcriptional responses varied across the injury period rather than increasing or decreasing uniformly.

**Figure 3:**
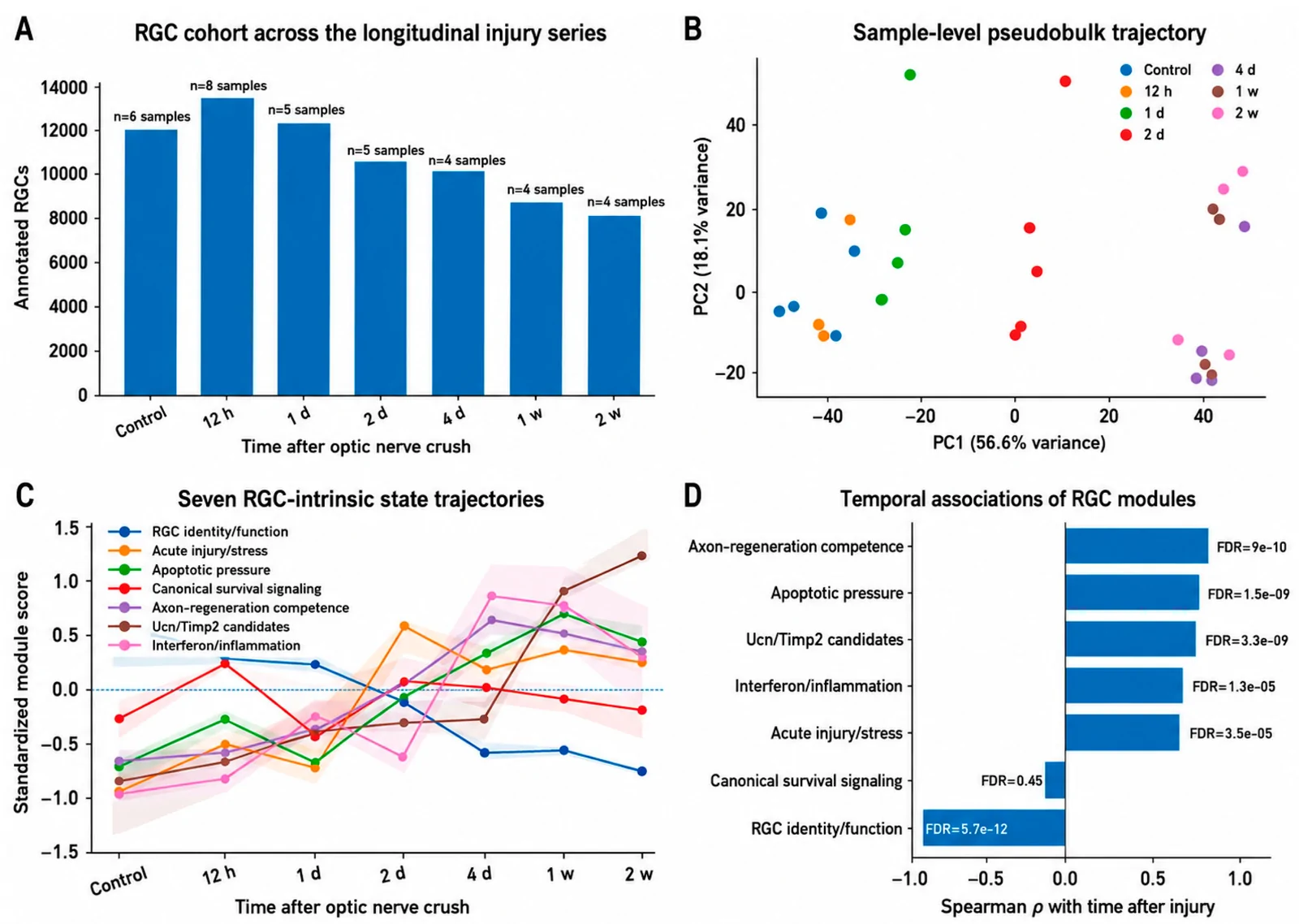
Longitudinal RGC transcriptomic trajectory after optic nerve crush. (**A**) Number of annotated RGCs and biological samples at each time point. (**B**) Principal-component representation of the 32 pseudobulk samples. (**C**) Temporal evolution of the seven standardized RGC modules. (**D**) Spearman correlations between module scores and time after injury.

Taken together, the RGC trajectory was not represented by a single unidirectional damage axis. Progressive loss of neuronal identity occurred simultaneously with increasing cellular stress, apoptotic pressure, inflammatory signaling, and regeneration-associated transcriptional activity. This coexistence of degeneration and attempted repair supported the use of separate state variables rather than a single aggregate injury score. Figure 3 integrates the evidence underlying this longitudinal RGC-state representation. Panels A and B show the biological-sample composition and the strong organization of pseudobulk expression along the post-injury trajectory, whereas Panels C and D demonstrate that neuronal-identity loss, injury-associated responses, and repair-associated transcription evolve with distinct temporal directions and magnitudes.

### 3.2. The Retinal Environment Showed an Early Reactive and Trophic Response Followed by Complement-Associated Hazard

The seven GSE241268 whole-retina samples contained 81,318 cells before study-specific quality control. A total of 72,985 cells, corresponding to 89.8% of the downloaded cohort, satisfied the conservative filtering criteria and were retained for environmental-state construction [6,17].

The retained cells were assigned to broad retinal compartments using transparent marker-panel scoring. Müller glia, microglial/myeloid cells, endothelial cells, RGCs, and other neuronal and non-neuronal populations were identified for the purpose of constructing compartment-specific pseudobulk profiles. These broad assignments were used as analytical groupings and were not interpreted as revised official annotations of the source dataset.

The environmental modules displayed a non-monotonic early injury response. At 12 h after optic nerve crush, the retinal environment showed the strongest combined Müller-cell reactivity, microglial activation, and loss of microglial homeostasis. This early reactive state was accompanied by a transient increase in Müller-cell trophic-support signaling.

Because only one biological sample was available at 12 h, this observation was treated as a descriptive temporal anchor rather than an independently replicated effect. Nevertheless, the coordinated direction of several environmental modules indicated a pronounced early response in the source trajectory.

At 24 h, the composite environmental-hazard state decreased relative to the 12 h condition and fell below the baseline-referenced level in the normalized trajectory. Microglial activation remained elevated, however, indicating that reduced aggregate environmental hazard did not correspond to a complete return to homeostasis.

At 48 h, Müller reactivity and complement-associated signaling increased again. In parallel, the Müller trophic-support module declined relative to its early 12 h increase. This pattern indicated a transition from an early mixed reactive–supportive response toward a later state characterized by complement activity and reduced trophic support.

Cytokine/interferon and vascular-activation modules varied between biological samples and time points. Given the limited number of biological samples, these variables were retained as contextual environmental signals and were not interpreted as definitive evidence of specific cell-type mechanisms. Extracellular matrix remodeling also contributed to the environmental state but did not follow a simple monotonic trajectory over the first 48 h.

**Figure 4:**
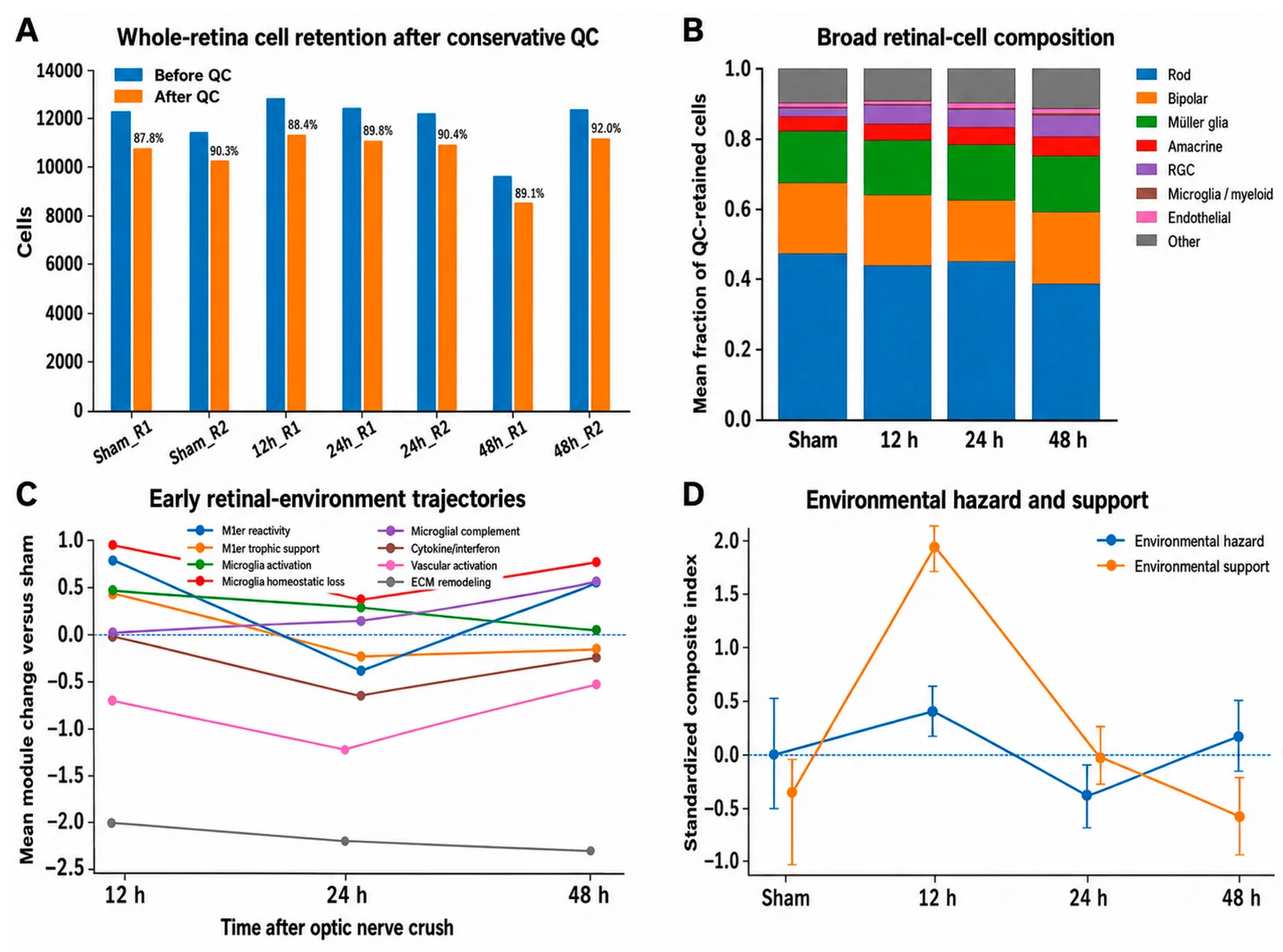
Early retinal environment response after optic nerve crush. (**A**) Numbers of cells before and after quality control for each biological sample. (**B**) Broad retinal-cell compartments used for pseudobulk construction. (**C**) Standardized Müller, microglial, vascular, and extracellular matrix module trajectories. (**D**) Composite environmental-support and environmental-hazard indices at sham, 12 h, 24 h, and 48 h.

Overall, the early retinal response combined potentially protective and potentially adverse processes. Müller-cell trophic support and reactive glial signaling increased concurrently, while microglial activation was accompanied by loss of homeostatic characteristics. The later rise in complement-associated hazard further demonstrated that the retinal environment could not be represented adequately by a single inflammatory variable. Figure 4 summarizes the complementary retinal environment trajectory. Panels A and B document the retained cellular material and broad retinal compartments used for environmental-state construction, whereas Panels C and D show the coexistence of early reactive and trophic responses followed by increasing complement-associated hazard, supporting the use of multiple environmental variables rather than a single inflammatory score.

### 3.3. RGC-Intrinsic and Retinal Environment States Were Temporally Asynchronous

Temporal fusion of the seven RGC modules and eight retinal environment modules revealed that the neuronal and environmental responses did not reach their maximum deviations at the same time.

The strongest aggregate environmental response occurred at 12 h. At this point, Müller reactivity, microglial activation, microglial homeostatic loss, and trophic-support signaling were simultaneously elevated. In contrast, the composite RGC injury-risk state continued to increase beyond 12 h and reached its highest early-trajectory value at 2 days.

The RGC repair-capacity index also changed over time. It remained below its baseline-referenced midpoint at control and 12 h but moved toward positive values by 2 days. This increase occurred while RGC identity continued to decline and apoptotic pressure remained elevated. Therefore, increased regeneration-associated transcription did not correspond to restoration of neuronal health. Instead, it represented an attempted repair response developing within an increasingly injured neuronal population.

The fused trajectory consequently contained at least three partially dissociable processes: an early environmental reaction, progressive neuronal injury, and delayed activation of regeneration-associated transcriptional programs. Their temporal separation provided the biological rationale for retaining RGC-intrinsic and environmental modules as distinct controller inputs.

This result also supported the use of sequential rather than static decision making. An action selected solely from the early environmental state could differ from an action selected later, when environmental hazard had changed but neuronal injury had progressed. Similarly, the appearance of regeneration-associated competence did not necessarily justify immediate regeneration-oriented intervention if acute hazard or irreversible-injury risk remained high.

The observed asynchrony was descriptive. GSE137398 and GSE241268 were generated from different animals, experimental designs, and sequencing preparations. Accordingly, the fused trajectory did not establish causal signaling between environmental cells and RGCs, nor did it reconstruct direct cell–cell communication. It provided a temporally aligned, biologically interpretable state representation for the computational benchmark.

**Figure 5:**
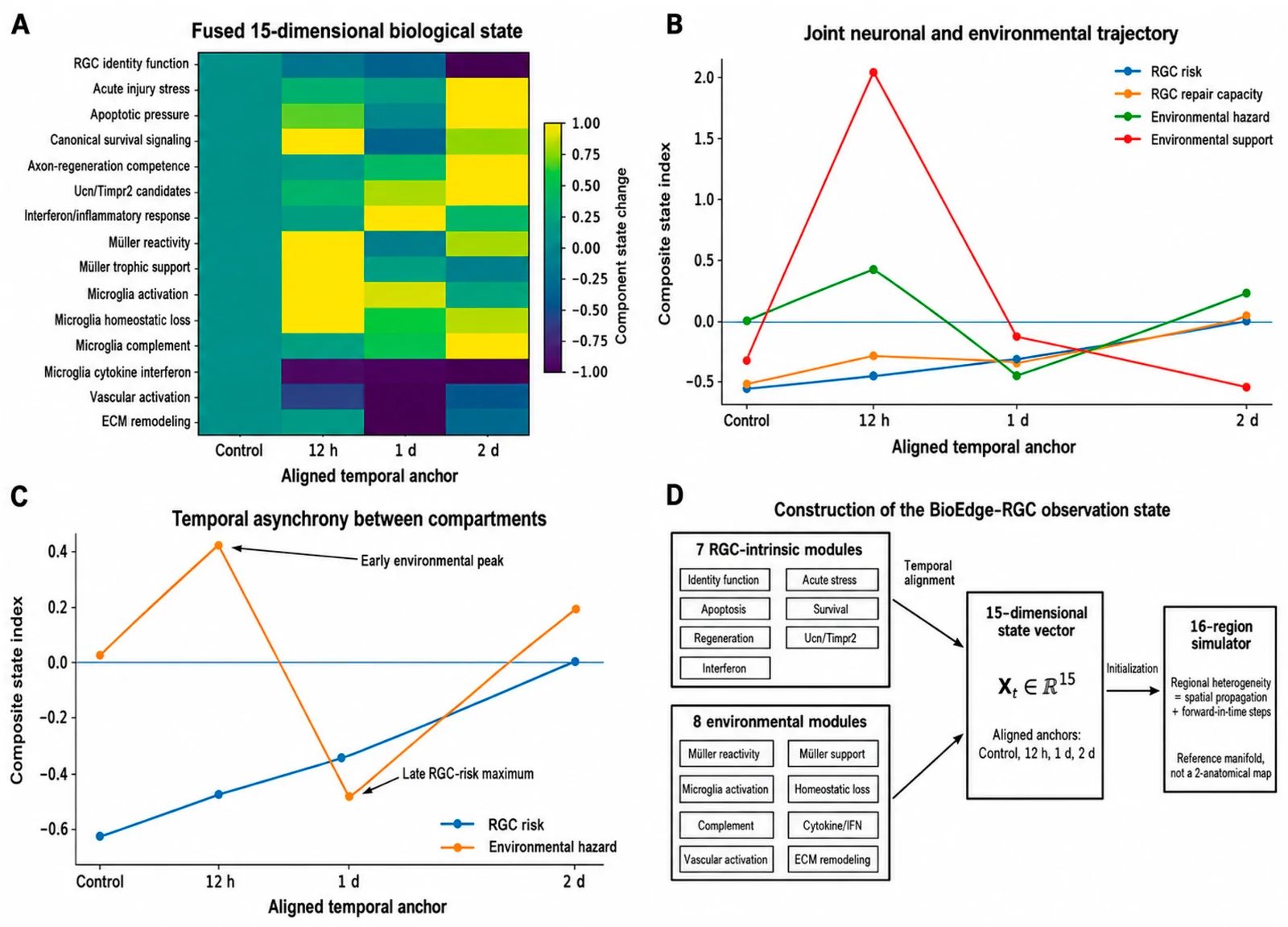
Temporal fusion of RGC-intrinsic and retinal environment states. (**A**) Seven RGC and eight environmental modules aligned at baseline, 12 h, 1 day, and 2 days. (**B**) Composite RGC-risk, RGC-repair-capacity, environmental-hazard, and environmental-support indices. (**C**) Temporal separation between the early environmental peak and the later RGC-risk maximum. (**D**) Construction of the 15-dimensional biological reference state used by the simulator.

The resulting 15-dimensional trajectory was used as the transcriptomics-informed temporal reference for initialization of the 16 simulated tissue regions. Regional perturbations, hidden susceptibility, spatial propagation, and process noise were subsequently introduced by the simulator and should not be interpreted as directly observed features of the transcriptomic datasets. Figure 5 illustrates how the independently derived neuronal and environmental trajectories were brought together without assuming paired multimodal measurements. Panels A and B show their alignment and composite indices, Panel C highlights the temporal separation between the early environmental response and the later RGC-risk maximum, and Panel D shows how these complementary signals form the 15-dimensional reference state subsequently used by the simulator.

#### An Independent Dnmt3a-Perturbation Dataset Provided Limited Plausibility Support for the RGC State Orientation

In GSE229033, 6 of the seven transferred RGC modules changed in the prespecified favorable direction in Dnmt3a-deficient RGCs relative to injured controls. The only nonconcordant module was Axon-regeneration competence, which showed a small change in the opposite direction. The strongest directionally concordant individual change was observed for apoptotic pressure, which decreased by 0.518 standardized-score units in the Dnmt3a-cKO group (bootstrap 95% interval, −0.893 to −0.094; Hedges’ g = −1.30).

The equal-weight oriented composite difference was 0.238 standardized-score units (bootstrap 95% interval, −0.061 to 0.533; Hedges’ g = 0.80). Because the composite interval included zero and each group contained only three biological samples, the analysis was interpreted as limited direction-and-effect-size plausibility support rather than confirmatory evidence of a regenerative treatment effect.

This external perturbation analysis provided an orthogonal plausibility check for the orientation of the RGC state variables. It was not interpreted as experimental validation of BioEdge-RGC intervention effects, transition dynamics, controller-selected actions, or controller performance. Complete sample-level and module-level results are provided in Supplementary Figure S1 and Supplementary Table S4.

### 3.4. The MPC Expert Generated a Strong Multi-Step Reference Policy

The candidate-constrained MPC expert was evaluated against no intervention and a fixed time-dependent protocol on the held-out test episodes. The same initial latent states and process-noise realizations were used for all controllers.

Without intervention, the mean five-step episode objective was 0.4773, the mean final simulated health was 0.5306, and irreversible failure occurred in 77.45% of episodes. These results confirmed that the simulated injury dynamics generated substantial progressive deterioration in the absence of control.

The fixed protocol substantially improved all three outcomes. Its mean objective increased to 0.6963, final health increased to 0.7079, and the failure rate decreased to 6.36%. Therefore, even a non-adaptive schedule was effective when its predefined actions were broadly compatible with the assumed injury dynamics.

The MPC expert achieved the strongest reference performance. Its mean episode objective was 0.7182, with an empirical 2.5th–97.5th percentile range of 0.3556–0.9239. Mean final health reached 0.7327, and the irreversible-failure rate decreased to 2.91%.

Compared with the fixed protocol, the MPC expert increased the mean objective by 0.0220 and reduced the failure rate by 3.45 percentage points. This corresponded to a relative failure reduction of approximately 54%. The advantage resulted from repeated state-dependent allocation rather than from a larger intervention budget, because both strategies were limited to five actively treated regions at each decision step.

The MPC expert was not treated as a mathematically optimal oracle. It evaluated a restricted set of feasible candidate allocations over a three-step horizon and operated without knowledge of future stochastic disturbances. Nevertheless, it provided a stronger and more adaptive reference policy than either uncontrolled progression or the fixed schedule.

**Figure 6:**
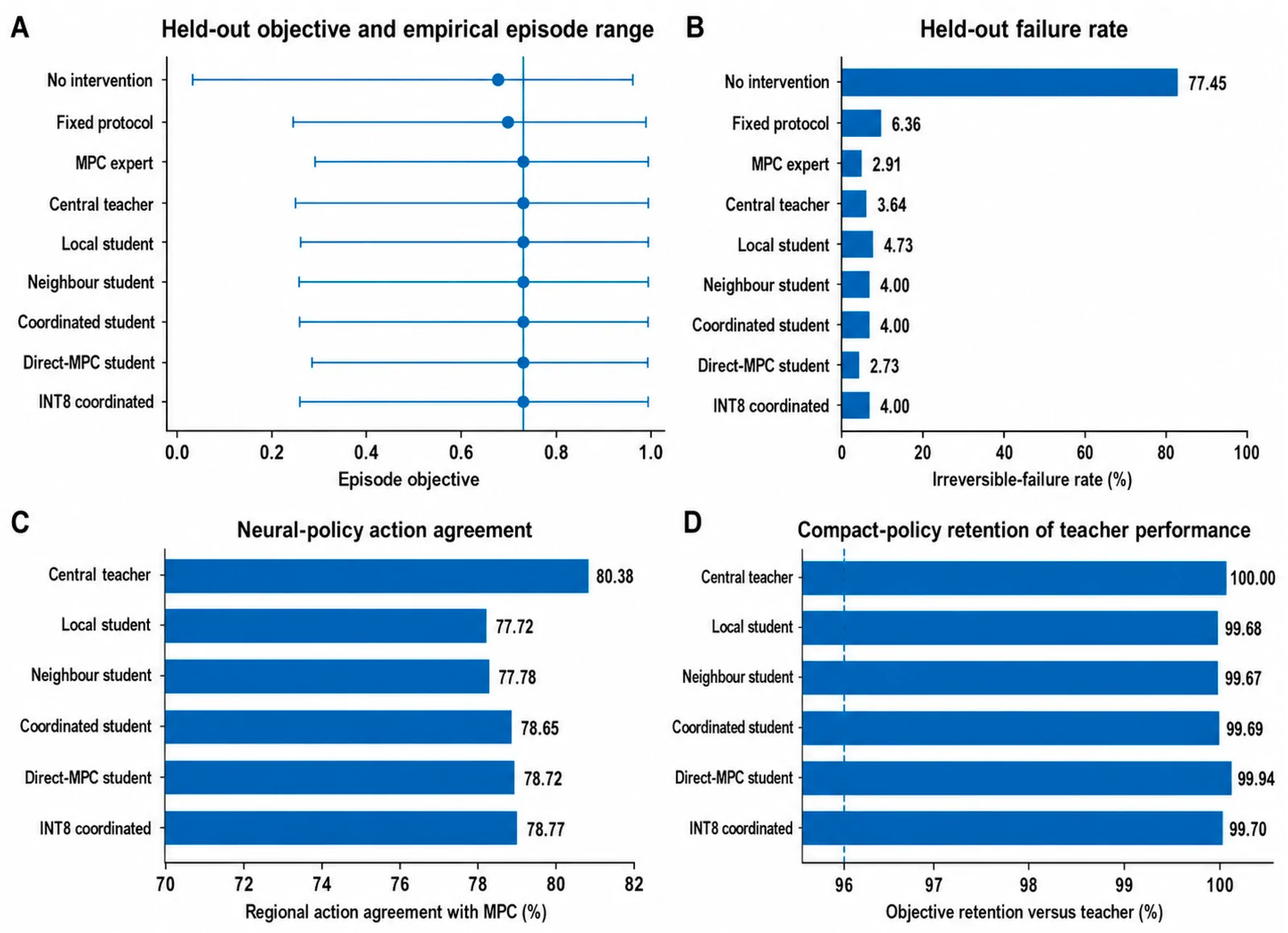
Primary held-out controller benchmark. (**A**) Mean five-step episode objective and empirical 2.5th–97.5th percentile range for the reference and neural controllers. The ranges represent variation among simulated episodes and are not confidence intervals for biological outcomes. (**B**) Irreversible-failure rate. (**C**) Regional action agreement of the neural controllers with the MPC reference controller. (**D**) Objective retention relative to the central teacher. Retention ratios should be interpreted together with the absolute objective differences reported in Table 5. The dashed line marks the predefined 96% coordinated-student retention criterion.

Figure 6A,B places these reference-controller results in the context of the complete held-out benchmark: the progressive improvement from no intervention to the fixed protocol and MPC is evident for both episode objective and irreversible failure. The corresponding held-out reference-controller metrics are reported in Table 4.

### 3.5. The Central Teacher Preserved the MPC Policy

The central neural teacher received the complete 16-region latent state, current decision step, and normalized intervention budget. It was trained to reproduce both the regional action classes and the relative priority of the five regions selected by the MPC expert.

On held-out episodes, the central neural teacher achieved a mean objective of 0.7175, compared with 0.7182 for the MPC reference controller. The absolute mean-objective difference was therefore −0.0007, corresponding to 99.90% performance retention under the ratio-based metric. Mean final simulated health was 0.7335, while the irreversible-failure rate was 3.64%.

The teacher reproduced 80.38% of the regional MPC action labels. Exact action agreement was therefore lower than objective retention, indicating that several alternative action allocations produced similar closed-loop outcomes within the simulator. The policy could preserve the MPC objective without copying every regional decision exactly.

The ranking head allowed the teacher to order regions under the five-region intervention budget. During final neural execution, the highest-probability active class among classes 1–3 was identified for each region, and the five regions with the highest priority scores were assigned those active classes; all remaining regions received no intervention. Thus, the teacher learned not only the preferred action class but also the relative allocation structure required by the constrained control problem.

The small difference between the teacher and the MPC expert satisfied the predefined teacher-approximation criterion of at least 97%. The central teacher was therefore considered an adequate neural approximation of the receding-horizon reference policy and was used as the source of soft action probabilities and regional-priority targets for student training.

Figure 6C,D further shows that near-complete objective retention did not require exact reproduction of every regional MPC action, distinguishing closed-loop performance preservation from action-by-action imitation.

### 3.6. Compact Regional Policies Approximated the Reference Controllers

All compact regional policies achieved mean objectives within 0.0024 of the central neural teacher despite receiving less information during neural inference and using smaller neural networks.

The local ranked student, which observed only the imputed state and availability mask of the corresponding region, achieved a mean objective of 0.7152. This represented an absolute difference of −0.0023 relative to the central teacher and corresponded to 99.68% performance retention under the ratio-based metric. Its mean final simulated health was 0.7322, and its irreversible-failure rate was 4.73%.

Adding compressed information from the two neighbouring regions produced a mean objective of 0.7151. The neighbour-aware student differed from the central teacher by −0.0024 and retained 99.67% of teacher performance. Its mean final simulated health was 0.7309, and its irreversible-failure rate was 4.00%.

The coordinated ranked student additionally received a compact tissue-level broadcast. It achieved a mean objective of 0.7153, corresponding to an absolute difference of −0.0022 and 99.69% performance retention relative to the central teacher. Its mean final simulated health was 0.7311, and its irreversible-failure rate was 4.00%. Regional action agreement with the MPC reference controller was 78.65%, compared with 77.72% for the local student and 77.78% for the neighbour-aware student.

A coordinated model with the same architecture was also trained directly from MPC-generated labels without teacher-probability or teacher-priority supervision. This direct-MPC student achieved a mean objective of 0.7170, corresponding to an absolute difference of −0.0005 and 99.94% retention relative to the central teacher. Its irreversible-failure rate was 2.73%.

The direct-MPC student slightly outperformed the hybrid-distilled coordinated student in both mean objective and failure rate. Consequently, the present results do not demonstrate that knowledge distillation was superior to direct behavioural cloning when the complete set of MPC-generated training labels was available. Distillation should instead be interpreted as a successful policy-transfer mechanism that preserved teacher behaviour in a compact model. Held-out performance and absolute objective differences for the neural controllers are summarized in Table 5.

Overall, the compact regional policies preserved the closed-loop performance of the central teacher despite reduced access to simulated tissue information and lower model complexity (Figure 6). All policies were subject to the same five-region budget-arbitration rule. The comparisons therefore evaluated differences in the information available during neural inference and in the resulting regional predictions, rather than differences in enforcement of the global intervention budget.

### 3.7. Nonlocal Context Reduced Failure but Did Not Improve the Mean Objective

The coordinated ranked student achieved a mean objective of 0.715287, compared with 0.715234 for the local ranked student. The mean paired episode-level difference was therefore

$$∆J_{coord\text{-}local}=0.000053$$

This difference represented approximately 0.007% of the local-student mean objective and was negligible in practical terms within the present simulator. It was also substantially smaller than the predefined minimum advantage of +0.008. The criterion intended to demonstrate a meaningful mean-objective benefit from coordinated information was therefore not met.

The coordinated student nevertheless reduced the irreversible-failure rate from 4.73% for the local student to 4.00%. This represented an absolute reduction of 0.73 percentage points and a relative failure reduction of 15.4%, which narrowly exceeded the predefined 15% criterion.

The neighbour-aware student also achieved a failure rate of 4.00%. Consequently, the observed reduction in failure could not be attributed uniquely to the compact tissue-level broadcast. Within the held-out test distribution, access to compressed information from adjacent regions was sufficient to achieve the same failure-rate reduction as the coordinated architecture.

The coordinated architecture increased regional action agreement with the MPC reference controller from 77.72% for the local student to 78.65%. However, this increase did not translate into a meaningful improvement in the mean episode objective. The local biological state, decision step, remaining intervention budget, and ranking-aware training may have provided sufficient information to approximate most of the required regional allocation policy.

These findings define the principal claim boundary of the regional-policy comparison. The results support the feasibility of transferring a globally generated MPC policy to compact regional neural models, but they do not establish that a compact tissue-level broadcast is indispensable for preserving the mean simulated objective. The more limited conclusion is that nonlocal information reduced simulated failure relative to local-only inference, while neighbour information alone achieved the same failure rate as the coordinated architecture.

### 3.8. Compact Messaging Reduced Communication, While Dynamic INT8 Quantization Reduced Model Storage

Centralized teacher execution required a modeled communication volume of 5520 bytes per five-step episode. This accounting represented transmission of the complete regional state to the centralized controller and return of the corresponding regional decisions.

The local architecture required 400 bytes per episode, corresponding to a 92.75% reduction relative to the centralized baseline. The neighbour-aware architecture required 1000 bytes and reduced communication by 81.88%.

The coordinated codec required 1080 bytes per episode. It therefore reduced the modeled communication volume by 80.43% while retaining both neighbour summaries and a compact global broadcast.

**Figure 7:**
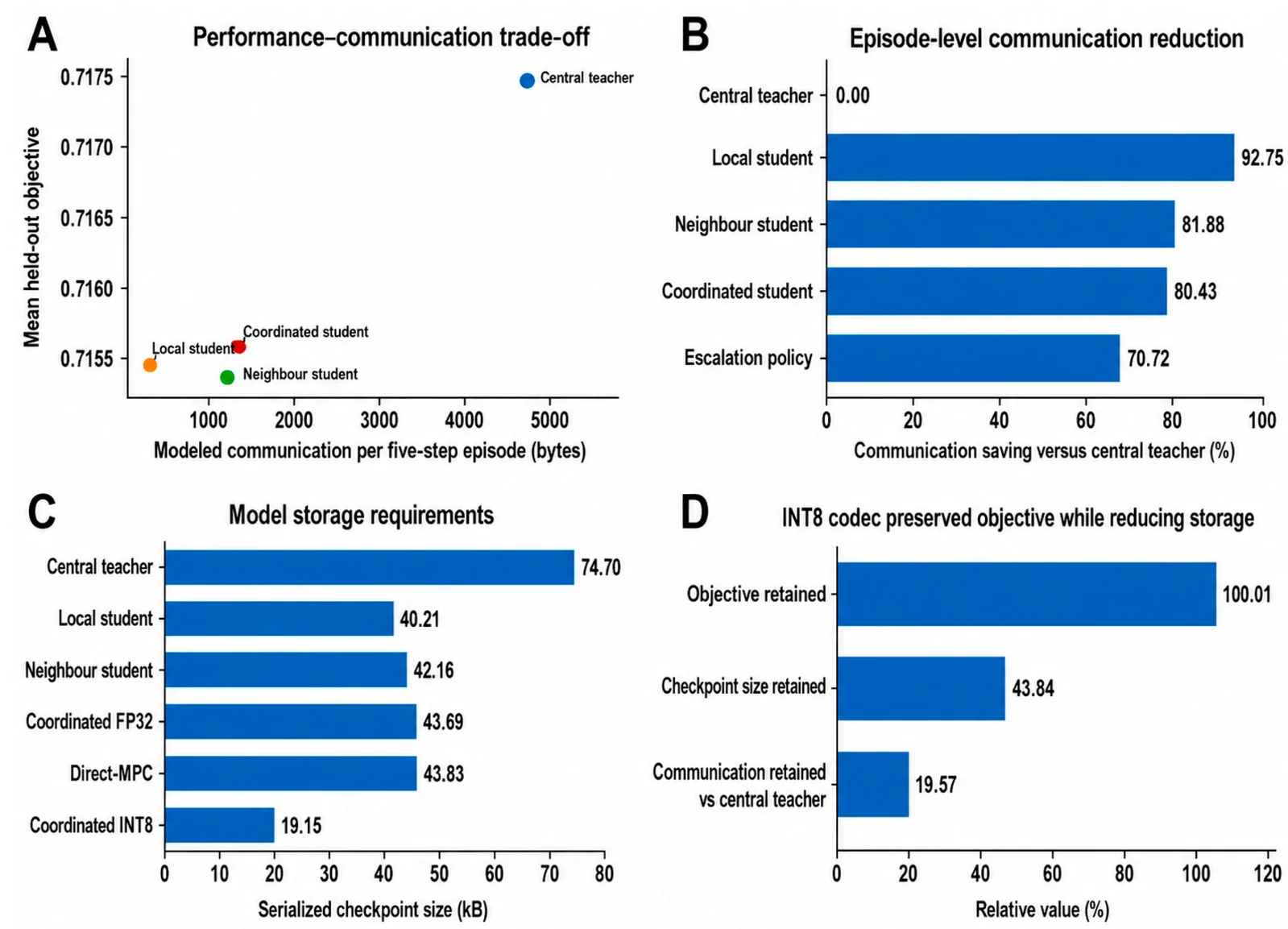
Model and communication efficiency. (**A**) Held-out objective as a function of modeled communication volume per five-step episode. (**B**) Communication saving relative to centralized teacher execution. (**C**) Serialized checkpoint size. (**D**) Relative objective retention, checkpoint-size retention, and communication retention for the INT8 coordinated implementation. Communication values are software-accounting quantities rather than physical energy measurements.

The resulting performance–communication trade-off is shown in Figure 7A,B. The panels show that all compact regional architectures occupy a markedly lower-communication regime than the centralized teacher while remaining tightly clustered in objective performance; the local model minimizes communication most strongly, whereas the neighbour-aware and coordinated models retain additional nonlocal context.

The INT8-coded coordinated controller achieved a mean objective of 0.71537, compared with 0.71529 for the FP32 coordinated model. Its performance retention relative to FP32 was 100.01%, and its failure rate remained unchanged at 4.00%. The difference was numerically negligible and should not be interpreted as superiority of the quantized controller.

The FP32 coordinated student contained 9933 trainable parameters and occupied 43.69 kB as a serialized checkpoint. Dynamic INT8 conversion reduced its serialized size to 19.15 kB, corresponding to a reduction of approximately 56%. The central teacher contained 17,853 parameters and occupied 74.70 kB.

Model-storage requirements and the FP32–INT8 comparison are summarized in Figure 7C,D. Panels C and D separate two distinct engineering effects of quantization: checkpoint storage was substantially reduced, whereas objective retention remained essentially unchanged and communication requirements were unaffected.

A separate single-threaded CPU implementation benchmark measured one complete 16-region neural forward pass. Median inference latency was approximately 101.6 μs for the central teacher, 55.0 μs for the local student, 56.1 μs for the neighbour-aware student, and 55.2 μs for the FP32 coordinated student. The dynamically quantized INT8 model required approximately 136.6 μs.

Thus, dynamic INT8 model quantization reduced checkpoint storage but did not reduce latency or alter the modeled communication volume in the tested software environment. The additional quantization and operator-dispatch overhead exceeded the computational saving expected from the small network. These latency values are implementation- and hardware-specific and should not be interpreted as estimates of biological, implant, or molecular execution time.

Thus, compact edge execution preserved the reference-policy objective while substantially reducing both communication volume and model storage (Figure 7).

Model complexity, serialized size, and modeled communication requirements are summarized in Table 6.

### 3.9. Edge Policies Remained Robust to Missing Sensors and Out-of-Distribution Injury

Progressive missing-sensor robustness was evaluated on the fixed severe-injury OOD episode set while preserving the same underlying latent episodes across missingness levels. Sensor masking therefore changed only the observations available to the controller and did not alter injury severity, state-transition dynamics, or process-noise realizations.

With complete observations, the coordinated student achieved a mean objective of 0.6026, final health of 0.6363, and a failure rate of 23.27% on the predefined robustness episode set.

**Figure 8:**
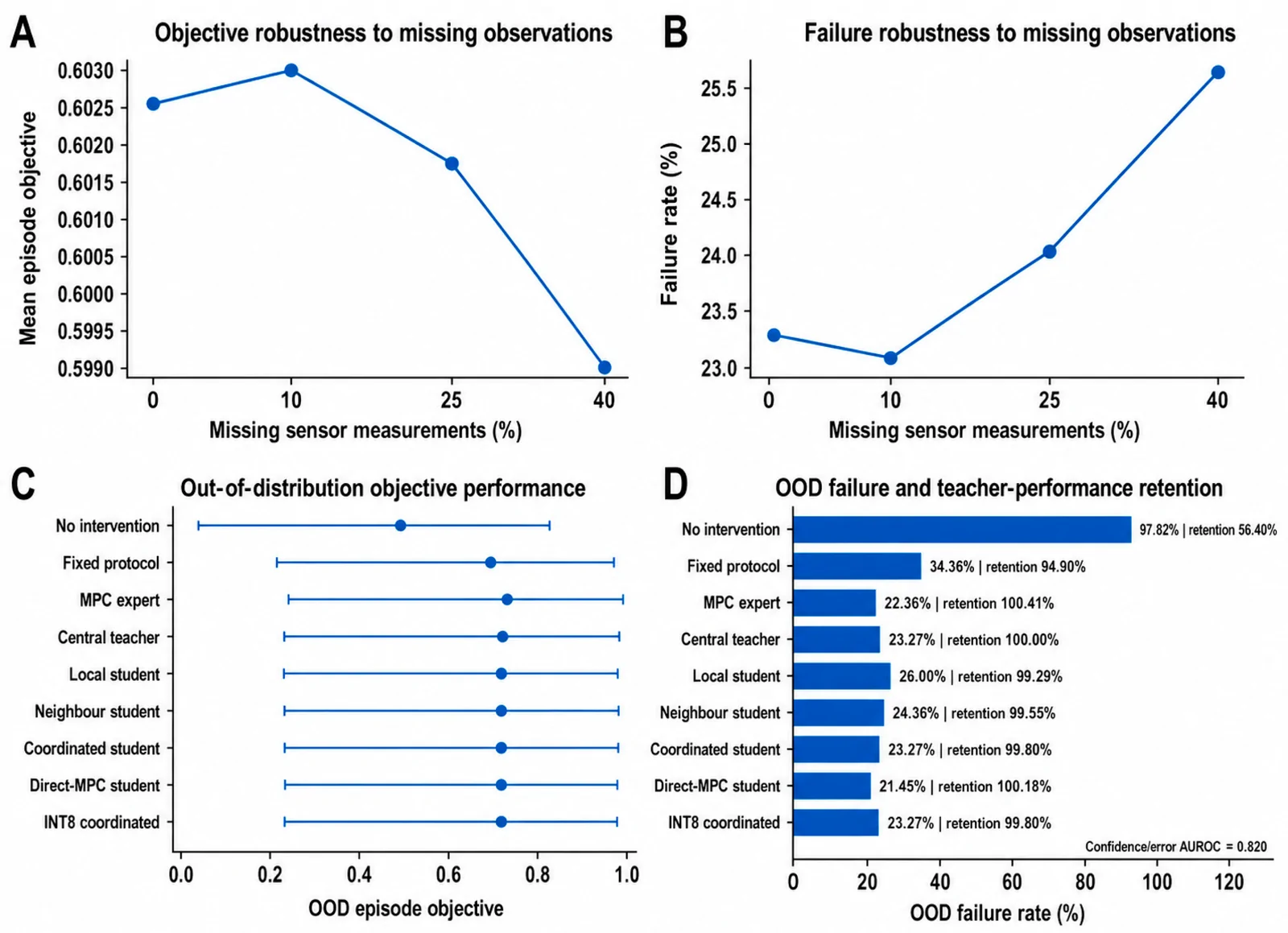
Robustness to missing observations and out-of-distribution injury. (**A**) Coordinated-student objective with 0%, 10%, 25%, and 40% sensor missingness. (**B**) Corresponding irreversible-failure rates. Sensor masking changed only the controller observations; the latent episodes remained unchanged. (**C**) Out-of-distribution episode objective and empirical 2.5th–97.5th percentile range. (**D**) OOD failure rate and objective retention relative to the central teacher.

At 10% sensor missingness, the mean objective was 0.6030 and the failure rate was 23.09%. At 25% missingness, the objective remained 0.6017 and the failure rate was 24.00%. Even at 40% missingness, the objective decreased only to 0.5990, while final health remained 0.6355 and failure increased to 25.64%. Robustness to progressive sensor missingness is shown in Figure 8A,B. One can see from this figure that objective performance remains nearly flat through moderate observation loss, while the failure rate increases only modestly at the highest missingness level, indicating gradual rather than abrupt degradation. The corresponding progressive-missingness results are reported in Table 7.

Performance was therefore nearly stable between 0% and 25% missingness and degraded modestly at 40%. The small fluctuation at 10% missingness was within simulator variability and was not interpreted as a beneficial effect of sensor loss. Importantly, the corrected experiment did not generate artificial changes in the latent simulated tissue when sensors were masked.

Under out-of-distribution injury conditions, no intervention produced a mean objective of 0.3406 and a failure rate of 97.82%. The fixed protocol achieved an objective of 0.5731 and failure rate of 34.36%, whereas the MPC expert achieved 0.6064 and 22.36%, respectively.

The central teacher achieved a mean OOD objective of 0.6039, retaining 99.59% of MPC performance. The local student achieved 0.5997 with a failure rate of 26.00%. The neighbour-aware student improved the objective to 0.6012 and reduced failure to 24.36%.

The coordinated student achieved an OOD objective of 0.6027 and a failure rate of 23.27%. It retained 99.80% of teacher performance and 99.39% of MPC performance. The INT8-coded coordinated model produced nearly identical results, with an objective of 0.60274 and the same 23.27% failure rate. Figure 8C,D extends this comparison to the predefined severe-injury distribution and shows that the relative ordering of the principal adaptive controllers is largely preserved despite the expected reduction in absolute performance.

The direct-MPC coordinated student achieved the strongest neural OOD result, with an objective of 0.6050 and a failure rate of 21.45%. This again indicated that direct expert supervision remained at least as effective as hybrid distillation in the full-data setting.

These results indicate gradual rather than catastrophic degradation under incomplete sensing and more severe simulated injury conditions (Figure 8). Detailed out-of-distribution controller performance is reported in Table 8.

### 3.10. Comparative Controller Conclusions Were Preserved Under Local Parameter Perturbations

The local parameter-sensitivity analysis comprised the baseline-parameter condition and eight one-factor-at-a-time perturbation conditions evaluated on the same fixed cohort of 550 episodes. Central-teacher retention relative to the scenario-specific MPC expert ranged from 99.79% to 100.20%, while coordinated-student retention relative to the central teacher ranged from 99.47% to 99.84%. Values slightly above 100% reflected small mean differences on the fixed simulated cohort and were not interpreted as evidence that the neural model was superior to the MPC expert.

The coordinated-minus-local mean-objective difference ranged from −0.001380 to +0.000811. It remained below the previously defined +0.008 meaningful-difference threshold in all nine parameter conditions. The architecture-matched direct-MPC student achieved a mean objective at least as high as that of the hybrid-distilled coordinated student in all nine conditions.

The coordinated-versus-local failure-rate difference was less stable. Relative failure reduction ranged from −6.0% to 33.3% and reached the 15% practical threshold in three of the nine conditions. Under the stricter failure definition, the coordinated failure rate was 9.64%, compared with 9.09% for the local student. Therefore, the analysis supported the robustness of compact policy transfer, the absence of a meaningful mean-objective advantage from coordination, and the lack of superiority of hybrid distillation over direct MPC supervision. It did not support a parameter-robust failure-rate advantage of the coordinated architecture.

Complete scenario definitions, controller-level outcomes, episode-level results, and structural-comparison summaries are provided in Supplementary Table S3.

### 3.11. BioEdge-RGC Enabled Reproducible Interactive Simulation

**Figure 9:**
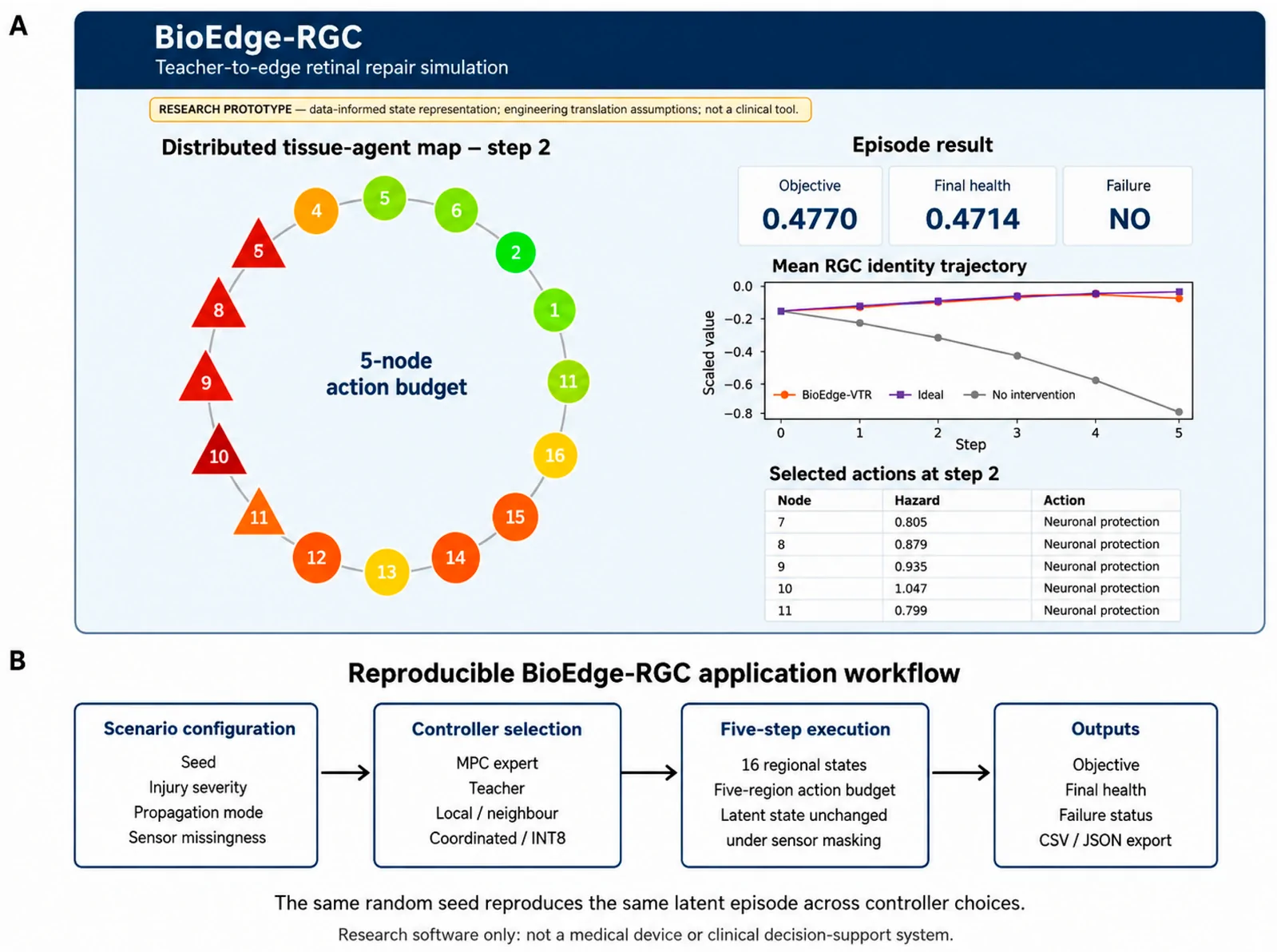
BioEdge-RGC interactive research application. (**A**) Complete application interface showing the 16-region tissue-agent map, five-region action budget, episode-level objective, final simulated health, failure status, RGC-identity trajectory, and selected regional actions. (**B**) Reproducible workflow linking scenario configuration, controller selection, five-step execution, visualization, and CSV/JSON export. The same random seed reproduces the same latent episode across controller choices. The application is research software and not a medical device or clinical decision-support system.

The trained controllers were incorporated into the BioEdge-RGC interactive research application. The application executes complete five-step episodes in a local browser interface and uses the locked V3.2 model checkpoints. The complete user interface and the corresponding reproducible workflow are shown in Figure 9.

Each generated scenario contains 16 interconnected tissue regions. A user-defined random seed controls initialization of regional injury severity, hidden susceptibility, propagation pattern, process noise, and sensor availability. Reusing the same seed therefore reproduces the same latent episode across different controller selections.

The application supports both standard and out-of-distribution injury conditions. The propagation mode can be selected as clockwise, counter-clockwise, multifocal, or automatically generated. The proportion of missing biological measurements can also be varied without altering the underlying latent tissue state.

Users can select among no intervention, the fixed protocol, the MPC expert, the central teacher, the local student, the neighbour-aware student, the coordinated distilled student, the direct-MPC student, and the INT8-coded coordinated controller.

The complete interface displays the 16-region tissue-agent map, the five-region intervention budget, regional hazard states, measurement availability, selected action classes, and the actions assigned at each decision step. Episode-level outputs include the cumulative objective, final simulated tissue health, irreversible-failure status, and the temporal evolution of RGC identity and environmental hazard (Figure 9A).

The complete five-step regional action schedule can be exported as a CSV file. A detailed JSON export contains the latent states, incomplete controller observations, sensor-availability masks, action probabilities, regional priorities, selected actions, and episode-level outcomes. Scenario configuration, controller selection, five-step execution, visualization, and data export form a reproducible local workflow, as summarized in Figure 9B.

BioEdge-RGC serves as a research and software-demonstration environment for comparing globally informed and edge-executed control policies. It does not recommend interventions for animals or patients, does not estimate the efficacy of a specific treatment, and is not a medical device or clinical decision-support system.

## 4. Discussion

### 4.1. Principal Findings

The present study examined whether a globally planned, multi-step tissue-control policy could be transferred to compact regional agents operating with incomplete sensing and compressed communication. BioEdge-RGC combined a biologically informed 15-dimensional injury-state representation, a spatial multi-region simulator, a candidate-constrained MPC expert, a central neural teacher, and three progressively more informed edge-student architectures.

The principal result was that the globally informed control policy could be compressed with minimal loss of closed-loop performance. The central teacher retained 99.90% of the MPC objective, while the coordinated edge student retained 99.69% of teacher performance. This transfer was achieved despite replacing full access to all 16 regional states with imputed local observations, explicit availability masks, compressed neighbour summaries, and a limited global broadcast.

The communication requirement of coordinated execution was reduced from 5520 bytes for centralized teacher execution to 1080 bytes per five-step episode, corresponding to an 80.43% reduction. INT8 coding preserved 100.01% of the FP32 coordinated objective while decreasing serialized model size. The value slightly above 100% represents negligible numerical variation and should not be interpreted as an improvement caused by quantization.

The results also establish important negative findings. The mean-objective difference between the coordinated and local students was only +0.000053, substantially below the predefined meaningful-difference threshold of +0.008. Although coordination reduced the failure rate by 15.4% relative to local-only execution, the neighbour-aware student produced the same failure rate as the coordinated model. Consequently, the results do not demonstrate that a global broadcast was indispensable.

Furthermore, the direct-MPC student performed at least as well as the hybrid-distilled student. The study therefore demonstrates successful policy transfer and compression, but it does not establish that knowledge distillation is superior to direct behavioural cloning when complete MPC labels are available.

### 4.2. Biological State Representation and Its Interpretation

The biological component of BioEdge-RGC was intended to provide a structured injury-state representation rather than a mechanistic model of optic nerve repair. The RGC-intrinsic trajectory showed progressive loss of neuronal identity together with increasing stress, apoptotic pressure, inflammatory signaling, and regeneration-associated transcription. This coexistence is consistent with the observation that injured RGC populations can simultaneously contain degenerative and attempted repair programs rather than progressing along a single scalar damage axis [1]. The transcriptomic analysis therefore supported the selection and temporal organization of the state coordinates but did not validate the assumed regional transition dynamics or the effects assigned to the abstract intervention classes.

The whole-retina dataset added a distinct representation of the early tissue environment. Müller reactivity, microglial activation, homeostatic loss, trophic support, complement signaling, vascular activation, and extracellular matrix remodeling followed non-identical trajectories. The early environmental response was strongest around 12 h, whereas the composite neuronal risk continued to increase and reached its maximum later in the fused early trajectory. This temporal asynchrony supported the decision to preserve the neuronal and environmental variables as separate controller inputs.

The 15-dimensional representation was preferable to a single injury score because states with similar aggregate severity could differ in their balance of acute stress, environmental hazard, neuronal identity, and regeneration-associated competence. Such distinctions created a more meaningful sequential-control problem in which the selected action could depend on the composition, rather than only the magnitude, of injury.

Nevertheless, the fused state was descriptive. GSE137398 and GSE241268 were generated from different animals, tissue preparations, and experimental protocols [1,6]. Their alignment at common time points did not create paired multi-omic observations and did not reconstruct causal signaling between RGCs and surrounding cells. The simulator therefore used transcriptomic data to define plausible observation coordinates, not to infer experimentally validated state-transition laws.

The independent GSE229033 analysis modestly strengthened the biological interpretation of the RGC state representation without changing the scope of the study. Six of seven unmodified modules changed in the prespecified favorable direction following RGC-specific Dnmt3a deficiency, although the small sample size and bootstrap interval spanning zero for the oriented composite preclude confirmatory inference. The result therefore reduces, but does not eliminate, concern that the RGC module orientation reflects only idiosyncratic patterns in GSE137398. It does not establish that the simulator transition equations are biologically correct, that the abstract intervention-effect vectors reproduce Dnmt3a suppression, or that any controller-selected action would improve RGC survival or axonal regeneration.

The observed module orientation is also biologically compatible with the source Dnmt3a study, which associated Dnmt3a suppression with activation of neuronal-survival and axon-growth programs together with attenuation of apoptosis- and inflammation-associated networks [23]. More broadly, experimental optic-nerve studies have shown that axonal regrowth alone does not guarantee functional recovery; neural activity, target-specific reconnection, synaptic function, and effective conduction represent additional and partly separable requirements [24,25].

### 4.3. Compact Policy Learning Through Distillation and Direct MPC Supervision

MPC provides a natural framework for sequential decision making under state constraints, intervention costs, and finite resource budgets, but repeated online evaluation of candidate future trajectories can be computationally demanding [8]. In BioEdge-RGC, the candidate-constrained MPC reference controller was therefore used as a policy generator rather than as the intended model for regional edge execution.

Policy distillation has similarly been used to transfer expert decision policies into substantially smaller neural models, although preservation of closed-loop performance remains distinct from reproducing individual action labels [13].

The central neural teacher reproduced nearly the complete MPC objective while achieving approximately 80% regional action agreement. The difference between action agreement and objective retention is informative because it indicates that multiple regional action allocations could produce similar episode-level outcomes within the simulator. Exact reproduction of every MPC-selected regional action was therefore not required to preserve closed-loop performance.

The teacher architecture processed each region using a shared encoder and constructed global context through pooled regional representations. This permutation-equivariant structure prevented arbitrary regional indices from being learned as fixed biological identities and was appropriate for the exchangeable simulated tissue regions used in the present benchmark [21].

The regional-priority head was also important conceptually. The policy had to determine not only which action class was appropriate for each region, but also which five regions should receive active intervention when more regions were considered eligible. Action classification alone could not fully represent this constrained allocation problem. Pairwise ranking and priority regression therefore provided an explicit mechanism for approximating the ordering generated by the MPC reference controller under the intervention budget.

All compact regional policies achieved mean objectives within 0.0024 of the central teacher, indicating that the MPC-generated policy was highly compressible within the evaluated simulator. Much of the regional allocation structure could be inferred from the local injury state, decision step, remaining intervention budget, sensor-availability information, and, depending on the architecture, compact neighbour or tissue-level summaries.

Hybrid knowledge distillation supplemented the hard MPC-generated labels with soft teacher action probabilities and continuous regional-priority targets [11]. However, the architecture-matched direct-MPC student achieved a slightly higher mean objective and a lower irreversible-failure rate than the hybrid-distilled coordinated student. The present benchmark therefore does not demonstrate that teacher-based soft supervision improved closed-loop performance when the complete set of MPC-generated labels was available. Both training strategies supported compact policy transfer, but direct MPC supervision was at least as effective under the evaluated full-label conditions.

This negative comparison does not imply that knowledge distillation is ineffective in other settings. Soft teacher supervision may provide an advantage when MPC queries are computationally expensive, when only a restricted number of hard expert labels is available, when labels are noisy or incomplete, when the teacher has access to privileged information unavailable to the student, or when uncertainty and relative regional priorities are insufficiently represented by a single hard action allocation. These conditions were not evaluated in the present study and require separate experiments.

Neural approximation and imitation of MPC policies have also been investigated as strategies for reducing online optimization cost while retaining behavior close to the reference controller [14,15].

### 4.4. What Coordination Added—And What It Did Not Add

The coordinated student received both compressed neighbour information and a compact global broadcast, whereas the local student received only its own incomplete regional observation. The coordinated model reduced failure from 4.73% to 4.00%, corresponding to a relative reduction of 15.4%. This indicates that nonlocal information could modify decisions in a subset of difficult episodes. The term coordination refers here to the additional neighbour and compact tissue-level information available during regional neural inference. All architectures used the same lightweight centralized arbitration procedure to enforce the five-region intervention budget. The comparison therefore did not test fully decentralized resource allocation. This distinction is consistent with the established separation between decentralized, distributed, and hierarchical MPC architectures, which differ principally in the availability of inter-controller communication and centralized coordination [26].

However, the mean-objective advantage was only +0.000053 and did not meet the predefined +0.008 threshold. The principal coordination hypothesis was therefore not supported for the mean objective. Moreover, the neighbour-aware student also produced a 4.00% failure rate, showing that the global summary was not uniquely responsible for the reduction.

Several explanations are possible. First, regional hazard and action suitability may have been strongly encoded in the local 15-dimensional state. Second, the intervention budget and decision time provided indirect information about the expected global allocation regime. Third, ranking-aware training may have allowed the local model to approximate population-level priorities from repeated simulated examples. Finally, the five-step horizon and 16-region ring may not have generated enough long-range dependence to require global coordination.

The defensible interpretation is therefore narrower than an assertion that tissue-wide communication is necessary. BioEdge-RGC demonstrates that limited nonlocal information can reduce simulated failure without materially changing the average objective. Whether global summaries become essential under longer horizons, larger tissue graphs, delayed propagation, asymmetric regional observability, or stricter budgets remains an open question.

Future evaluations should deliberately construct scenarios in which two regions have similar local states but require different actions because of distant hazards, future resource competition, or tissue-wide constraints. Such counterfactual cases would provide a stronger test of whether a global broadcast contributes information that cannot be reconstructed locally.

### 4.5. Communication Compression, Quantization, and Edge Feasibility

The communication results support the central engineering motivation of edge intelligence: placing compact computation near observations and actuators can reduce dependence on full centralized state transmission [10]. The coordinated architecture required 1080 bytes per episode compared with 5520 bytes for centralized teacher execution, while retaining 99.69% of teacher performance.

The local student achieved the largest communication reduction but lacked neighbour and global context. The neighbour-aware and coordinated models occupied an intermediate position, providing additional context while still transmitting less than one-fifth of the centralized volume. This creates a practical design space rather than a single universally optimal architecture.

Dynamic INT8 model quantization preserved the FP32 coordinated objective and reduced serialized checkpoint size, consistent with the role of integer quantization in compact neural inference [22]. However, dynamic INT8 conversion did not reduce latency on the tested workstation. The networks were small enough that quantization and operator-dispatch overhead outweighed the expected arithmetic saving.

Consequently, the present INT8 result supports storage and representation efficiency rather than faster execution. Actual edge deployment would require testing on the intended processor, microcontroller, neuromorphic device, or embedded accelerator. Static quantization, quantization-aware training, integer-native operators, and memory-transfer measurements may produce different results from dynamic desktop quantization.

The reported byte counts also represent a software communication model. They are not measurements of wireless energy, implant power, molecular diffusion, biological signaling, or transmission latency. The communication benefit should therefore be interpreted as an algorithmic reduction in represented information, not as a demonstrated reduction in physical energy consumption.

### 4.6. Robustness to Missing Observations and More Severe Simulated Injury, and Parameter Perturbations

The missingness experiment was designed so that sensor failure affected only controller observations. The underlying latent episodes remained unchanged across missingness levels. This distinction was necessary because modifying the latent state together with the observation mask would confound sensor robustness with changes in simulated injury severity.

The coordinated student remained nearly stable between 0% and 25% missingness. At 40% missingness, the objective decreased modestly and failure increased from 23.27% to 25.64%. The degradation was therefore gradual rather than catastrophic. Explicit availability masks and training-time sensor dropout probably contributed to this behaviour by allowing the model to distinguish measured and imputed values.

Performance also remained close to the teacher under the predefined out-of-distribution injury conditions. However, these conditions were generated by increasing severity, propagation, hidden susceptibility, and initial heterogeneity within the same simulator family. OOD robustness in this context does not establish transfer to another biological model, species, sequencing platform, or injury mechanism.

The local parameter-sensitivity analysis extended this robustness assessment to moderate misspecification of the simulator parameters. Across the baseline configuration and eight one-factor-at-a-time perturbation conditions involving action-effect magnitude, neighbour-propagation strength, process-noise scale, and failure thresholds, the compact neural policies remained close to the scenario-specific MPC expert. The coordinated-minus-local mean-objective difference remained below the predefined +0.008 threshold in all nine conditions, and direct MPC supervision remained at least comparable to hybrid distillation. However, the coordinated-versus-local failure-rate difference varied substantially across parameter conditions and did not consistently meet the 15% practical threshold. Thus, the principal objective-based conclusions were robust to local parameter perturbations, whereas the apparent failure-rate advantage of coordination was sensitive to the simulator assumptions and should not be interpreted as a stable architectural benefit.

The explicit use of availability masks is consistent with previous missing-data neural architectures showing that observation patterns can contain information distinct from the imputed numerical values themselves [27].

### 4.7. Role of the BioEdge-RGC Application

The interactive application converts the computational benchmark into an inspectable and reproducible research tool. The same random seed can be used to compare multiple controllers on an identical latent episode, while sensor missingness changes the observations without changing the underlying simulated tissue.

The application also exposes regional states, priorities, selected actions, objective trajectories, and failure outcomes rather than presenting only aggregate benchmark values. CSV and JSON exports permit individual episodes to be audited and reproduced outside the interface.

This transparency is particularly important for a simulated control system because similar average objectives can conceal different regional allocation patterns or failure mechanisms. The application can therefore support software validation, teaching, sensitivity testing, and preparation of prospective experimental protocols.

Its function remains limited to research demonstration. BioEdge-RGC does not accept patient measurements, predict clinical outcomes, recommend therapies, or model the effect of a named biological intervention. It should not be described as a medical device, autonomous treatment platform, or validated digital twin. Accordingly, future interoperability with standards such as HL7 FHIR or DICOM would constitute an engineering integration pathway rather than evidence that the present framework has achieved clinical validation or digital-twin status.

### 4.8. Limitations

Several limitations define the interpretation of the study.

First, the RGC and retinal environment datasets were independent and were aligned only by nominal time after injury. No paired animals, spatial transcriptomic coordinates, or within-sample neuronal–environment measurements were available. The fused state therefore represents temporal co-alignment rather than directly observed tissue interactions.

Second, the environmental dataset contained only one biological sample at 12 h. This time point was retained because it was important for early alignment, but its between-sample variability could not be estimated.

Third, the seven RGC and eight environmental modules were manually specified from interpretable gene panels. Alternative module definitions, normalization procedures, or scaling rules could alter the resulting state representation. Prospective comparison with pathway-level, latent-factor, and multimodal representations is needed.

Fourth, the regional topology, propagation dynamics, hidden susceptibility, intervention-effect vectors, objective weights, clipping limits, and failure thresholds were engineering assumptions. The positive controller results do not demonstrate that corresponding biological interventions would preserve RGCs or promote axonal regeneration.

Fifth, the 16-region ring was not an anatomical model of the retina, optic nerve head, or optic nerve. It provided a controlled topology for studying local propagation and budgeted allocation. Anatomically informed graphs would require spatial and structural data not included here.

Sixth, the neural policies were trained on episodes produced by the same simulator family used for evaluation. The held-out and OOD seeds were independent, but the underlying transition structure remained related to the training environment. Simulator-level generalization cannot substitute for biological external validation.

Seventh, the predefined coordination criterion for mean objective was not met. The study therefore does not demonstrate the necessity of global communication or superiority of the coordinated architecture over local execution.

Eighth, the direct-MPC student performed as well as or better than the distilled student. The results support compact policy learning but do not demonstrate a specific advantage of knowledge distillation over direct expert supervision.

Ninth, the regional models were not fully autonomous decentralized agents. Although neural inference was performed using architecture-specific local, neighbour, or compact global inputs, final enforcement of the five-region budget required a common arbitration layer. The present results therefore support regionalized edge inference with centralized budget enforcement rather than fully decentralized multi-agent control.

Tenth, communication volume, model size, and desktop latency are engineering proxies. No physical edge hardware, sensor network, implant, microfluidic system, or biological communication channel was tested.

Finally, no new animal experiments, axon counts, electrophysiological measurements, target reinnervation assessments, visual-function tests, or intervention-response data were obtained. Biological efficacy and safety remain entirely unvalidated.

### 4.9. Experimental Roadmap and Key Directions for Future Research

The key directions for future research are to move progressively from the present software benchmark toward experimentally measured state transitions, hardware-constrained distributed execution, and ultimately biologically validated closed-loop control. The next step should be prospective hardware-in-the-loop validation rather than further unconstrained simulator optimization. A retinal explant, RGC–glia co-culture, or microfluidic axotomy platform could be divided into local sensing and actuation zones corresponding to the simulated regional agents.

Longitudinal imaging, live/dead reporters, calcium activity, transcriptional reporters, neurite extension, and electrophysiological measurements could provide repeated within-sample observations. Controlled perturbations would then allow estimation of state–action–next-state transitions instead of assuming intervention-effect vectors.

A minimum experimental comparator set should include no intervention, a fixed schedule, centralized adaptive control, local edge control, neighbour-aware control, and coordinated control. Direct-MPC imitation and distilled policies should also be evaluated separately because the current software benchmark did not demonstrate a distillation advantage.

The primary endpoints should include RGC survival, directed axon extension, functional activity, unsafe actions, intervention burden, inference latency, physical communication volume, energy use, and failure to act under sensor loss. Model selection and success criteria should be specified before experimental evaluation.

The BioEdge-RGC hypothesis should be considered unsupported if compact execution fails to preserve centralized performance, if communication savings disappear on real hardware, if nonlocal messages provide no benefit in deliberately coordination-dependent tasks, or if adaptive policies increase unsafe biological outcomes. Such prospective falsification criteria are essential before any progression toward in vivo autonomous control.

## 5. Conclusions

BioEdge-RGC demonstrates, within a transcriptomics-informed multi-region simulator, that a candidate-constrained MPC policy can be approximated by a central neural teacher and transferred to compact regional models operating with incomplete observations and compressed communication. The teacher retained 99.90% of the MPC objective, and the coordinated student retained 99.69% of teacher performance while reducing modeled communication by 80.43%. INT8 coding preserved FP32 performance and reduced model storage.

The comparative results impose important boundaries on this contribution. Coordination did not produce a meaningful mean-objective advantage over local execution, neighbour-aware and coordinated execution achieved the same primary-benchmark failure rate, and direct MPC supervision performed at least as well as hybrid distillation. Across nine local parameter conditions, compact policy transfer and the principal objective-based comparisons were preserved, but the coordinated-versus-local failure-rate advantage was not robust to the evaluated simulator assumptions.

The independent GSE229033 analysis provided limited external plausibility support for the RGC state orientation: six of seven transferred modules changed in the predefined favorable direction, but the oriented composite bootstrap interval included zero. This result supports biological coherence of the state representation only and does not validate the transition equations, abstract intervention effects, MPC decisions, or controller efficacy.

The supported contribution is therefore the reproducible transfer and compression of a globally planned multi-step policy into small, missingness-aware regional models under a fixed intervention budget. BioEdge-RGC does not validate optic nerve repair, biological treatment efficacy, a digital twin, or clinical decision support. Prospective state–action–next-state perturbation data and hardware-in-the-loop experiments remain necessary before biological or translational meaning can be assigned to the controller outputs.

## Figures and Tables

**Table 1:** Public transcriptomic datasets used in the BioEdge-RGC study.

Dataset	Biological Contribution	Time Points Used	Samples and Observations	Role in BioEdge-RGC
GSE137398	RGC-intrinsic response after optic nerve crush	Control, 12 h, 1, 2, 4, 7, and 14 days	32 biological samples; 76,646 annotated RGCs	Construction of seven RGC modules and the longitudinal neuronal trajectory
GSE241268	Early whole-retina microenvironment after optic nerve crush	Sham, 12, 24, and 48 h	Seven biological samples; 81,318 cells before and 72,985 cells after QC	Construction of eight glial, immune, vascular, and extracellular matrix modules
GSE248537	RGC-enriched *Oprm1* overexpression after optic nerve crush	Five-day injury control and five-day *Oprm1* overexpression	One biological sample per arm	Source-study context only; excluded from state construction and the primary AI benchmark.
GSE229033	RGC-specific Dnmt3a perturbation after optic nerve crush	2 days after optic nerve crush	Three control and three Dnmt3a-cKO bulk RGC samples	Independent transcriptomic plausibility assessment of the seven transferred RGC modules; excluded from state construction and controller development

**Table 2:** RGC-intrinsic state modules used in BioEdge-RGC.

Module	Genes
RGC identity/function	*Rbpms*, *Pou4f1*, *Pou4f2*, *Pou4f3*, *Slc17a6*, *Thy1*, *Sncg*, *Tubb3*, *Stmn2*, *Snap25*, *Syt1*
Acute injury/stress	*Atf3*, *Jun*, *Junb*, *Fos*, *Egr1*, *Ddit3*, *Hspa1a*, *Hspa1b*, *Gadd45a*, *Trib3*
Apoptotic pressure	*Bax*, *Bbc3*, *Pmaip1*, *Casp3*, *Casp7*, *Apaf1*, *Bak1*, *Bid*, *Dapk1*, *Trp53inp1*
Canonical survival signaling	*Bcl2*, *Bcl2l1*, *Mcl1*, *Akt1*, *Akt3*, *Stat3*, *Socs3*, *Nfe2l2*, *Hif1a*, *Xbp1*, *Hspa5*
Axon-regeneration competence	*Gap43*, *Sprr1a*, *Sox11*, *Klf6*, *Klf7*, *Ecel1*, *Tubb6*, *Stmn1*, *Map1b*, *Gadd45a*
Neuroprotective candidates	*Ucn*, *Timp2*
Interferon/inflammatory response	*Stat1*, *Irf7*, *Isg15*, *Ifit1*, *Ifit2*, *Ifit3*, *Cxcl10*, *Ccl2*, *Oasl1*, *Rsad2*

**Table 3:** Environmental modules used in the 15-dimensional state.

Environmental Module	Cellular Compartment	Principal Genes
Müller reactivity	Müller glia	*Gfap*, *Vim*, *Stat3*, *Socs3*, *C3*, *Apoe*, *Clu*, *Lcn2*, *Serpina3n*, *S100a6*
Müller trophic support	Müller glia	*Bdnf*, *Cntf*, *Lif*, *Igf1*, *Fgf2*, *Vegfa*, *Gdnf*, *Ntf3*
Microglial activation	Microglia/myeloid	*Aif1*, *Tyrobp*, *Trem2*, *Cst7*, *Lpl*, *Apoe*, *Itgax*, *Cd68*, *Ctsb*, *Ctsd*
Microglial homeostatic loss	Microglia/myeloid	Negative score of *P2ry12*, *Tmem119*, *Cx3cr1*, *Sall1*, and *Hexb*
Microglial complement	Microglia/myeloid	*C1qa*, *C1qb*, *C1qc*, *C3*, *C4b*, *Cfh*
Microglial cytokine/interferon	Microglia/myeloid	*Il1b*, *Tnf*, *Ccl2*, *Cxcl10*, *Ifit1*, *Ifit3*, *Isg15*, *Stat1*, *Irf7*
Vascular activation	Endothelial cells	*Icam1*, *Vcam1*, *Sele*, *Selp*, *Kdr*, *Klf2*, *Klf4*, *Cxcl10*
ECM remodeling	Pooled non-neuronal environment	*Mmp2*, *Mmp9*, *Mmp12*, *Timp1*, *Timp2*, *Fn1*, *Col1a1*, *Col3a1*, *Serpine1*

**Table 4:** Held-out performance of the reference controllers.

Controller	Mean Objective	Gain vs. No Intervention	Empirical 2.5th–97.5th Percentile Range	Mean Final Health	Failure Rate
No intervention	0.4773	Reference	0.0847–0.8570	0.5306	77.45%
Fixed protocol	0.6963	+0.2190	0.2880–0.9176	0.7079	6.36%
MPC reference controller	0.7182	+0.2409	0.3556–0.9239	0.7327	2.91%

Note: The fixed protocol recovered most of the objective improvement achieved by MPC relative to no intervention, whereas the additional advantage of MPC was observed through state-dependent allocation and a lower failure rate.

**Table 5:** Held-out performance and absolute objective differences in the neural controllers.

Controller	Mean Objective	Absolute Difference vs. Teacher	Retention vs. Teacher (%)	Mean Final Health	Failure Rate (%)	MPC Action Agreement (%)
Central teacher	0.7175	Reference	100.00	0.7335	3.64	80.38
Local ranked student	0.7152	−0.0023	99.68	0.7322	4.73	77.72
Neighbour-aware student	0.7151	−0.0024	99.67	0.7309	4.00	77.78
Coordinated ranked student	0.7153	−0.0022	99.69	0.7311	4.00	78.65
Coordinated direct-MPC student	0.7170	−0.0005	99.94	0.7314	2.73	78.73

Note: Absolute objective differences were calculated relative to the central neural teacher. Negative values indicate a lower mean objective than the teacher. Retention ratios should be interpreted together with these absolute differences because the ratio depends on the numerical scaling of the simulated objective.

**Table 6:** Model complexity and communication requirements.

Architecture	Parameters	Serialized Size (kB)	Bytes per Episode	Communication Saving (%)
Central teacher	17,853	74.70	5520	Reference
Local ranked	9069	40.21	400	92.75
Neighbour ranked	9549	42.16	1000	81.88
Coordinated ranked FP32	9933	43.69	1080	80.43
Coordinated direct MPC	9933	43.83	1080	80.43
Coordinated dynamic INT8	9933	19.15	1080	80.43

**Table 7:** Robustness of the coordinated student to observation missingness under severe OOD injury.

Missing Sensors (%)	Mean Objective	Mean Final Health	Failure Rate (%)
0	0.6026	0.6363	23.27
10	0.6030	0.6366	23.09
25	0.6017	0.6364	24.00
40	0.5990	0.6355	25.64

Note: The same latent OOD episodes were used at all missingness levels; only controller observations and availability masks were changed. The absolute values are therefore not directly comparable with those of the primary held-out benchmark.

**Table 8:** Out-of-distribution controller performance.

Controller	Mean Objective	Retention vs. Teacher (%)	Mean Final Health	Failure Rate (%)
No intervention	0.3406	56.40	0.4076	97.82
Fixed protocol	0.5731	94.90	0.6132	34.36
MPC expert	0.6064	100.41	0.6398	22.36
Central teacher	0.6039	100.00	0.6381	23.27
Local ranked student	0.5997	99.29	0.6368	26.00
Neighbour-aware student	0.6012	99.55	0.6363	24.36
Coordinated ranked student	0.6027	99.80	0.6365	23.27
Coordinated direct-MPC student	0.6050	100.18	0.6367	21.45
Coordinated INT8 codec	0.6027	99.80	0.6365	23.27

## Data Availability

The public mouse transcriptomic datasets used in this study are available through the NCBI Gene Expression Omnibus under accessions GSE137398, GSE241268, GSE248537, and GSE229033. The derived 15-dimensional temporal reference anchors, biological-module definitions, retained simulator and controller parameters, locked neural checkpoints, primary aggregate evaluation outputs, verification scripts, and BioEdge-RGC interactive application are provided in Supplementary File S1 and Supplementary Tables S1 and S2. The parameter-sensitivity configuration, fixed seeds, run-level outputs, controller summaries, structural-comparison results, and analysis code are provided in Supplementary File S2 and Supplementary Table S3. The processed GSE229033 module scores, gene-coverage audit, effect-size estimates, bootstrap summaries, analysis code, and generated figure are provided in Supplementary File S3, Supplementary Table S4, and Supplementary Figure S1. The original V3.2 model-training script and its optimizer and loss hyperparameters, the raw CPU-latency benchmark script, and the component-level communication byte ledger were not retained. No patient-level data or newly generated animal data were used.

## Supplementary Materials

The following supporting information can be downloaded at https://www.mdpi.com/article/10.3390/brainsci16090941/s1: Supplementary File S1: BioEdge-RGC V3.2 reproducibility package containing the retained simulator and MPC implementation, locked neural-policy checkpoints, temporal reference anchors, aggregate evaluation outputs, interactive application, verification scripts, integrity manifest, and execution instructions; Supplementary File S2: Google Colab notebook, standalone Python script, fixed seeds, configuration files, run-level outputs, controller summaries, and figures for the local parameter-sensitivity analysis; Supplementary File S3: Analysis code, sample-level module scores, gene-coverage audit, effect-size tables, bootstrap summaries, and figure-generation outputs for the independent GSE229033 transcriptomic plausibility assessment; Supplementary Table S1: Complete definitions of the RGC-intrinsic and retinal environment biological modules; Supplementary Table S2: BioEdge-RGC V3.2 simulator, controller, intervention-effect, objective-function, neural-architecture, communication, and evaluation parameters; Supplementary Table S3: Local one-factor-at-a-time parameter-sensitivity analysis of the locked BioEdge-RGC V3.2 controllers; Supplementary Table S4: Independent GSE229033 Dnmt3a-perturbation assessment of the prespecified BioEdge-RGC modules; Supplementary Figure S1: Independent transcriptomic plausibility assessment of the prespecified RGC modules using GSE229033.
