# Supplementary material for: BioEdge-RGC: Compact Neural Policy Transfer from Model-Predictive Control in a Transcriptomics-Informed Optic Nerve Injury Simulation
Source: Brain Sci. 2026 Sep 2;16(9):941. doi: 10.3390/brainsci16090941 (PMC13604355; doi:10.3390/brainsci16090941)
Supplement: Supplementary file 1 [file brainsci-16-00941-s001.zip › Supplementary_File_S1_BioEdge_RGC_Reproducibility_Package.zip!/Supplementary_File_S1_BioEdge_RGC/BIOEDGE_RGC_APP_V1/BioEdge_RGC_Demo.html]

BioEdge-RGC Interactive Demo

# BioEdge-RGC

Interactive teacher-to-edge retinal repair simulation — publication prototype

**Research prototype.** The biological state is data-informed, but action effects and transitions are engineering assumptions. This is not a diagnostic or treatment tool.

Scenario

Controller

Decision step0

Missing sensors

## Distributed tissue-agent map

No actionProtectionEnvironmentRegeneration

## Episode result

Objective

Final health

Failure

## Closed-loop trajectories

## Selected actions

| Node | Hazard | Action |
| --- | --- | --- |

BioEdge-RGC application demo. Full Python application in the accompanying package supports reproducible seeds, OOD scenarios, missingness, comparisons and CSV/JSON export.
